# Advance care planning for the severely ill in the hospital: a randomized trial

**DOI:** 10.1136/bmjspcare-2017-001489

**Published:** 2019-01-21

**Authors:** Tanja Krones, Ana Budilivschi, Isabelle Karzig, Theodore Otto, Fabio Valeri, Nikola Biller-Andorno, Christine Mitchell, Barbara Loupatatzis

**Affiliations:** 1 Head Clinical Ethics, University Hospital Zürich/Institute of Biomedical Ethics and History of Medicine University of Zürich, Zürich, Switzerland; 2 Psychologist, Clinical Ethics, University Hospital Zürich, Zürich, Switzerland; 3 Emergency specialist nurse, Clinical Ethics, University Hospital Zürich/Institute of Biomedical Ethics and History of Medicine University of Zürich, Zürich, Switzerland; 4 Social Worker and Intensive Care Nurse, Clinical Ethics, University Hospital Zürich/Institute of Biomedical Ethics and History of Medicine University of Zürich, Zürich, Switzerland; 5 Statistician, Institute of Primary Care, University of Zürich, Zürich, Switzerland; 6 Director of the Institute of Biomedical Ethics and History of Medicine, University of Zürich, Zürich, Switzerland; 7 Center for Bioethics, Harvard Medical School, Boston, Massachusetts, USA; 8 Palliative Care Physician, Palliative Care Unit, University Hospital Zürich, Zürich, Switzerland

**Keywords:** advance care planning, shared decision-making, decision aid, randomized trial, pragmatic trial, resuscitation, last place-of-care

## Abstract

**Objectives:**

To investigate the impact of advance care planning (ACP) including decision aids for severely ill medical inpatients.

**Methods:**

Single-centre randomised controlled trial at a Swiss university hospital. Patients were randomly assigned (1:1) to receive an extra consultation with the hospital social service or a consultation with in-house facilitators trained according to an internationally established ACP programme. Trial participants with the exception of the observers were fully blinded. 115 competent severely ill adults, their surrogates and their attending physicians were enrolled and followed for 6 months after discharge or 3 months after death. The patient’s wishes regarding resuscitation (primary outcome), last place of care and other end-of-life wishes were recorded. Knowledge and respect of the patient’s wishes by the surrogates and attending physician were monitored.

**Results:**

Compared with controls, 6 months after the intervention, fewer patients wished to be resuscitated or were undecided (p=0.01), resuscitation wishes were documented more frequently (89% vs 64%, p=0.02) and surrogates and/or attending physicians had greater knowledge of the patient’s wishes (62% vs 30%, p=0.01). Groups were not different with regard to wishes being fulfilled, with the exception of last place of care being achieved more frequently in the intervention group (29% vs 11 %, p=0.05).

**Conclusion:**

ACP including decision aids offered to severely ill medical inpatients leads to greater knowledge, documentation and respect of treatment and end-of-life wishes. Introducing ACP to these patients however may be too late for many patients. Early integration of ACP during the illness trajectory and a broader regional approach may be more appropriate.

## Introduction

Advance care planning (ACP) has attracted growing attention since the 1990s. ACP describes a structured interactive process involving patients, their loved ones and their care providers to plan future treatments that respect patients’ wishes and goals.^1 2^ Over the past 20 years the focus has shifted from completion of advance directives to effective professional communication promoting patient-centred goals-of-care discussions for future care. Several systematic reviews on the effectiveness of ACP strategies^3–5^ indicate that ACP interventions increase the number of advance directives (ADs) and do-not-attempt-to-resuscitate orders (DNAR). More complex ACP interventions improve the quality of end-of-life care and the concordance of patients’ preferences with care.^6^ Some studies, mostly of low quality,^7^ include encouraging patients to complete ADs or placing DNAR orders without professional communication such as ACP, which is contrary to the core definition of the concept.^1 2^ An additional form of ACP interventions focuses on delivery of information through evidence-based decision aids without offering professional ACP communication. Systematic reviews on ACP decision aids for future care^3 8–10^ suggest that patients have less decisional conflict and tend to favour less intensive care similar to the impact of decision aids for current treatment decisions,^11^ although studies in the palliative care context are still rare.^12^ In none of the complex interventions were the two concepts of ACP facilitation and ACP decision aids combined. However, both concepts are necessary for delivering patient-centred care and improving evidence-based patient choice for future treatments. Although experts in the field consider care consistent with goals as the most important outcome measure of ACP,^13^ this has not often been objectively measured. Most commonly this is inferred by asking surrogates if patients’ wishes were fulfilled, using proxies such as combined measures of wishes known and fulfilled, or reviewing documentation in medical charts as wish fulfilment without reporting how the patients’ wishes were evaluated^6^. ACP experts described this discrepancy as ‘a dilemma for our field of research, and potentially setting up a policy dilemma as well’^13^(page 7).

We report the outcomes of a patient, surrogate and physician-blinded parallel group randomised controlled trial on the impact of ACP on knowledge and fulfilment of concrete treatment wishes among severely ill adult medical inpatients. The ACP intervention was delivered by in-house non-physician ACP facilitators, who underwent a 2-day communication skills training in 2013 before study start (see online supplementary file 1) based on the Australian Respecting Patient Choices and the German beizeiten begleiten programmes. Both of these programmes are rooted in the US-American Respecting Choices initiative. Different from former programmes, the use of evidence-based ACP decision aids was included in the training and process. In 2010, only about 10% of the population >75 years had ADs in a Swiss national survey in 2010.^14^ In 2013, a national law declared AD as binding.^15^ Before our study began, there was no Swiss ACP programme in place. Our complex intervention was therefore introduced in an ‘ACP-naïve’ context such that a randomised controlled trial including blinding of patient and caregiver and concealment of allocation was possible. Our study (Multidisciplinary advance care planning and shared decision-making for end-of-life care trial, MAPS trial) is part of a national research programme on end-of-life care (NFP 67 end-of-life care). All data are open to be shared.

10.1136/bmjspcare-2017-001489.supp1 Supplementary data

## Methods

### Study design

The study team screened all patients once weekly on seven inpatient units participating in the study. Randomisation into intervention and control group was performed (1:1) by the clinical trial centre using a static unstratified multiblock computer randomisation to maintain balance across the seven units. The attending physician (general practitioner or specialist) in charge after discharge and the potential surrogate decision-makers of enrolled patients were also invited to participate. To blind patients and their surrogates, they were asked to participate in a study testing the impact of two different communication tools on discharge planning for severely ill patients on their quality of care received after discharge, without being informed of what to expect in each tool. Participating attending physicians were also blinded to the intervention by using the same information. Twelve patient surrogates declined to consent for the study and two patients withdrew shortly after randomisation. No further data were obtained for these 14 patient surrogate dyads after baseline assessment, but they were included into the analysis by multiple imputation (see below). Interviews after the interventions were conducted in both groups face-to-face or by telephone. Medical records were reviewed 6 months after discharge/intervention. Due to limited study resources, observers were not fully blinded since they screened patients for inclusion and interviewed patients after the interventions. Data monitoring and analysis were undertaken by blinded study team members on an intention-to-treat basis. In total, 115 patients were recruited between July 30 2013 and December 18 2014 to ensure a maximum follow-up of 9 months. Follow-up was completed in August 2015. Many patients were treated or died outside of the study hospital requiring further data collection, which was completed by September 2016.

### Study participants

Eligible patients were ≥age 18 admitted to internal medicine, oncology, radiation oncology, haematology, nephrology, dermatology or neurology wards at the University Hospital of Zurich. All patients were competent as assessed by their attending physicians and had sufficient German-language skills to follow the study procedures. Physicians assessed patients using a screening tool for palliative care needs, including the 12 months surprise question.^16^ All patients with a positive 12 months surprise question (ie, ‘I would not be surprised if my patient dies within the next 12 months’) were approached and invited to participate. The eligibility criteria were revised during the trial to permit inclusion of severely ill patients admitted to acute day wards of the hospital units and who were discharged within 2 days, if regular ambulatory follow-up was planned.

### Intervention

Patients randomised to the intervention group received ACP counselling from one of seven in-house ACP facilitators (ie, two social workers, one chaplain, two palliative care nurses and two patient counsellors, trained in ACP as summarised in online supplementary file 1) either during their hospital stay or during their next regular ambulatory visit(s). Patients randomised to the control group received counselling sessions by hospital social workers who had no training in ACP. The control group conversation addressed special needs of patients as identified by the patients themselves. The ACP facilitators offered a person-centred goals-of-care discussion to patients and their surrogates, according to the Respecting Patient Choices and beizeiten begleiten guidance, regarding their wishes in cases of future emergencies, possible incapacity for decision-making and deterioration of health status. Facilitators delivered up to three conversations, each lasting between 60 and 90 min. In addition, a 9 min video decision aid combining descriptions on general goals of care and cardiopulmonary resuscitation^17 18^ and a written decision aid library addressing resuscitation, intubation, dialysis, tube feeding and last place of care (in German only, available on request), based on previously published ones were offered during the consultation if desired.^19–26^ All decision aids except two^25 26^ had been included in the abovementioned systematic reviews on ACP decision aids, and all are registered in the Ottawa decision aid inventory.^27^ Patients’ wishes regarding their goals of care were documented in an AD if desired, which included an emergency form, developed by the beizeiten begleiten programme, adapted for Switzerland (in German only, available on request).

### Study assessment

Baseline data including socio-demographics, basic ACP-relevant information and criteria for palliative needs assessments as recommended^16^ were collected in all screened patients to assess possible differences between patients who were or were not included in the study. ACP-relevant questions were also posed to patients who did not want to participate in the study in a short questionnaire. The first assessment of the impact of the intervention was conducted face-to-face in the hospital directly after the ACP or the control group conversation, or by telephone after discharge. For patients enrolled on acute day wards, who received the ACP or control group conversations during their next ambulatory visits, initial data on the impact of the interventions were obtained after the conversations and endpoints were assessed 6 months after the interventions. In order to avoid contamination threats (ie, asking the patient about concrete end-of-life wishes before outcome assessment, which could trigger ACP conversations in the control group) and to capture the most recent preferences of patients close to death, patients’ concrete treatment wishes (ie, resuscitation, intubation, tube feeding, sedation, dialysis, intravenous fluids, antibiotics and last place of care) were assessed 6 months after discharge/intervention or after death. We independently interviewed patients, their surrogate decision-makers and attending physicians 6 months after discharge or intervention about current patients’ preferences regarding each of the eight measures mentioned above. In case of death, surrogates were interviewed after 3 months to assess congruency between surrogates and physicians regarding the patient’s presumed wishes, and if these were fulfilled. We constructed a tool to measure all possible categories of patients’ wishes and their fulfilment. Congruency and wish fulfilment were determined by comparing the patient wishes to the surrogate and physician responses and to the medical charts. All possible cases were captured (ie, the patients had clear wishes, preferred to leave the decision to surrogate or physician, were undecided or were unable to express their wishes to the study team due to severe illness 6 months after discharge or because they died before the follow-up interview). The process of assessment of congruency between patients, surrogates and physicians is outlined in table 1. Table 2 describes the assessment of end-of-life wish fulfilment through patient, surrogate and physician interviews and by chart review for documentation and fulfilment of concrete end-of-life wishes. As inconsistencies were possible between patient, surrogate and physician statements, and documented medical outcomes, we ranked data in terms of hierarchy with regard to achievement of wish fulfilment. The column succession from left to right in table 2 illustrates the hierarchy, according to which the wish fulfilment was determined in cases of incongruences from various data sources. Medical records were reviewed during and after follow-up. Data on mortality and wish fulfilment were documented until September 2016.

**Table 1:** Triple congruency measure of patients’ wishes (eg, do you/does the patient want to be resuscitated?)

Patient	Surrogate	Physician	Congruency
Yes	Yes	Yes	Yes
No	No	No	Yes
Yes	Yes	Missing	Yes
No	No	Missing	Yes
Yes	Missing	Yes	Yes
No	Missing	No	Yes
Missing	Yes	Yes	Yes
Missing	No	No	Yes
Leave decision	Leave decision	Leave decision	Yes
Leave decision	Leave decision	Missing	Yes
Missing	Leave decision	Leave decision	Yes
Leave decision	Missing	Leave decision	Yes
Yes	No	Yes	No
Yes	No	No	No
No	Yes	Yes	No
No	No	Yes	No
No	Yes	No	No
Yes	No	Missing	No
No	Yes	Missing	No
Yes	Missing	No	No
No	Missing	Yes	No
Missing	Yes	No	No
Missing	No	Yes	No
Not decided	Not decided/don’t know	Not decided/don’t know	No
Not decided	Not decided/don’t know	Missing	No
Not decided	Missing	Not decided/don’t know	No
Missing	Not decided/don’t know	Not decided/don’t know	No
Yes/no/leave decision/not decided/don’t know	Missing	Missing	Missing
Missing	Yes/no/leave decision/not decided/don’t know	Missing	Missing
Missing	Missing	Yes/no/leave decision/not decided/don’t know	Missing

Yes: patient agrees to (or surrogate or treating physician reports that the patient wants to) be resuscitated or intubated or dialysed, or tube fed or getting antibiotics or intravenous fluids or being sedated. Participants were also asked for their preferred last place of care.

No: patient refuses to (or surrogate or treating physician reports that the patient refuses to) be resuscitated or intubated or dialysed or tube fed or getting antibiotics or intravenous fluids or being sedated.

Leave decision: patients (or surrogate or treating physician reports that the patient wants to) leave the decision to the surrogate or the physician.

Not decided/don’t know: patients do not (or surrogate or treating physician reports that the patient did not) decide or do not know what to decide.

Last place of care: patient expressed ‘preferred last place of care’ (home, hospice/nursing home, hospital, intensive care unit), knowledge of surrogate and physician of patient’s preferred last place of care.

**Table 2:** Codes on wish fulfilment dependent on patients’ wishes, the clinical situation and data on wish fulfilment by different information sources

Patients’ wish	Was the patient in the situation?	Wish fulfilment (as stated by the patient)	Wish fulfilment (as stated by the surrogate)	Wish fulfilment (medical records)	Wish fulfilment (as stated by the physician)	Wish actually fulfilled?
Yes/no/don’t know/leave decision	No	–	–	–	–	Not applicable
Yes/no	Yes	Yes	–	–	–	Yes
Yes/no	Yes	Missing	Yes	–	–	Yes
Yes/no	Yes	Missing	Missing	Yes	–	Yes
Yes/no	Yes	Mising	Missing	Missing	Yes	Yes
Missing	Yes	Missing	Yes	–	–	Yes
Missing	Yes	Missing	Missing	Yes	–	Yes
Missing	Yes	Missing	Missing	Missing	Yes	Yes
Leave decision	Yes	–	Yes	–	–	Yes
Leave decision	Yes	–	Missing	–	Yes	Yes
Yes/no	Yes	No	–	–	–	No
Yes/no	Yes	Missing	No	–	–	No
Yes/no	Yes	Missing	Missing	No	–	No
Yes/no	Yes	Missing	Missing	Missing	No	No
Missing	Yes	Missing	No	–	-–	No
Missing	Yes	Missing	Missing	No	–	No
Missing	Yes	Missing	Missing	Missing	No	No
Leave decision	Yes	–	No	–	–	No
Leave decision	Yes	–	Missing	–	No	No
Leave decision	Yes	–	Missing	Yes	Missing	Unclear
Leave decision	Yes	–	Missing	No	Missing	Unclear
Don’t know	Yes	–	–	–	–	Unclear

Patients’ wish (yes/no): patients’ wish to be either resuscitated or intubated or dialysed, or tube fed or getting antibiotics or intravenous fluids or being sedated. The patient could also answer ‘I don’t know’ or ‘I leave the decision to surrogate or physician'.

Was the patient in the situation: determines whether the patient was resuscitated or intubated or dialysed, or tube fed or getting antibiotics or intravenous fluids or being sedated. If the patient was never in the situation, the wish fulfilment was recorded as not applicable.

Wish fulfilment (as stated by the patient): the documented wish fulfilment as stated by the patients had the highest priority for determining whether their wishes were fulfilled.

Wish fulfilment (as stated by the surrogate): if the wish of the patient was missing or if the patient left the decision to the surrogate, the statement of the surrogate had the highest priority for determining the patient’s wish fulfilment.

Wish fulfilment (medical records): if the patient’s and surrogate’s wish documentation was missing, the wish fulfilment was determined according to what was stated in the medical records. This could only be determined if the patient was in the situation and if the documentation was available.

Wish fulfilment (as stated by the physician): the wish fulfilment of the patient was determined according to the statement of the physician only in the situation where the wish was not stated by the patient or surrogate, and was not documented in the medical records. However, if the patient left the decision to the surrogate or physician, and the surrogate’s decision was missing, the physician’s statement had priority over what was documented in the medical records.

The wish regarding last place of care: this wish was determined only if the patient was dead at the moment when wish fulfilment was assessed.

The ‘–’ means that the statement was considered irrelevant to the determination of wish fulfilment.

Decisional conflict for future emergencies in both surrogates and patients was measured using the Decisional Conflict Scale (box 1).^28^

Depression and anxiety were measured using the Hospital Anxiety and Depression Scale (HADS)^29^ at 6 months after discharge or intervention. Hospitalisation rates and last place of care were documented. We assessed the impact of death on surrogates by the impact of event scale^30^ and the HADS^29^ 3 months after death. Differences between decisions taken, wish fulfilment and patient outcomes were analysed between the intervention and control groups.

Box 1 **Features of the Decisional Conflict Scale**

**Part A**

- Definition of index decision and options by research team.

**Part B**

- Decisional Conflict Scale-16 items, 5 factors (informed, values clarity, support, uncertainty, effective decision-making).

**Scores (0–100)**

- 0<25=low decisional conflict.
- 25–37.5=moderate decisional conflict.
- 37.5=high decisional conflict.

### Power calculation

Our power calculation is based on the Australian study^6^ using the primary outcome measures of wishes known to and, if applicable, respected by caregivers and as documented in medical records. As we defined wishes known by asking patients, surrogates and responsible physicians on concrete end-of-life wishes, monitored their fulfilment in the medical charts (see tables 1 and 2), and did not only use chart review regarding fulfilment of general goals of care wishes,^6^ we estimated a lower baseline (10% wishes known and respected) and smaller effect (30% wishes known and respected) for the MAPS study. To achieve 90% power with a certainty of 95% for the primary outcome measure of wishes of resuscitation being known and respected, we calculated a sample size of 89 patients in each study arm, for a total of 178 patients.

### Statistical analysis

Statistical analysis was performed according to Consolidated Standards of Reporting Trials (see online supplementary files 2; 3) using the intention-to-treat analysis strategy. Data were analysed at last available follow-up. Patients/surrogates who withdrew informed consent (n=14) after randomisation and from whom no data were obtained during follow-up were also included in the analysis according to intention-to-treat. ORs were calculated using logistic regression or Bayesian logistic regression. Primary outcomes were merged into one single variable to assess congruency (as described in tables 1 and 2). Given the intention-to-treat strategy, we applied two methods to deal with missing values. First, for the decisional conflict and HADS scales, an individual mean imputation was performed.^31^ Second, multiple imputation was used for all other outcomes including participants who withdrew their consent after baseline assessment.^32^ The two treatment groups were separately imputed and later merged into one data file for the analysis. Multiple imputation was performed with SPSS V.22, while multiple imputation pooling and outcome analysis was performed with R V.3.2.3 (for statistical methods including dual congruency seeonline supplementary file 2). According to our study design, we did not perform a posteriori tests and p value adjustment for multiple testing.^33^

10.1136/bmjspcare-2017-001489.supp2 Supplementary data

10.1136/bmjspcare-2017-001489.supp3 Supplementary data

## Results

**Figure 1:**
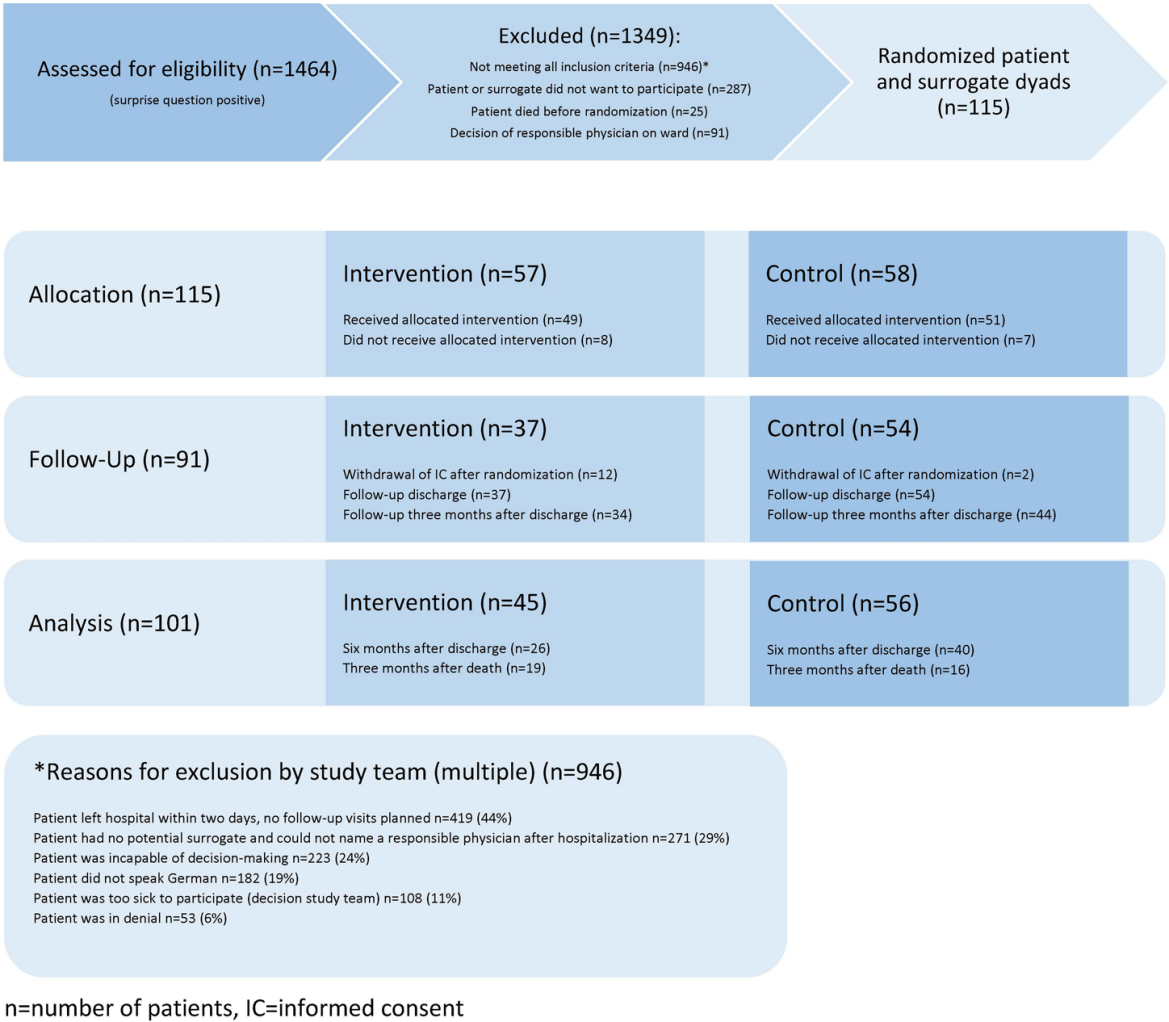
Study flowchart according to Consolidated Standards of Reporting Trials.

Of 1464 patients with a positive surprise question, 946 did not fulfil all inclusion criteria (figure 1).

Most non-participation was triggered by surrogates rather than patients, mostly due to acute stress precluding additional study procedures. Eighty-eight non-participant patients agreed to answer a short questionnaire. Primary criteria of possible unmet palliative care needs^16^ were assessed in 30 patients who declined to participate (non-participants) and 449 patients who were excluded (table 3). Compared with included patients, excluded patients had more complex care requirements and general decline in function, whereas participants had more acute difficult-to-control symptoms. Non-participants already had more established end-of-life wishes compared with participants (table 3). Participants were significantly younger and included more males and their (mostly) female surrogates. Patient ages ranged from 19 to 94 years (table 4). Although we screened oncology and non-oncology internal medicine wards, most patients had a primary diagnosis of cancer. We attempted to obtain follow-up data in all patients who did not withdraw informed consent, yet many missing values remained due to difficulties of reaching either patients, surrogates or responsible physicians in the ambulatory setting or obtaining medical records of outpatients.

**Table 3:** Baseline characteristics of all screened patients

	Included patients n (%) (n=115)	Non-participants n (%) (n=88)	Excluded patients n (%) (n=1261)	P values
Gender (male)	88 (77)	47 (53)	726 (58)	<0.001
Age mean (SD)	64.3 (15)	68.1 (14)	67.6 (15)	0.07
Frequent admissions (yes)	47 (41)	14 (47)	192 (43)	0.84
Difficult to control symptoms (yes)	96 (84)	20 (67)	330 (74)	0.05
Complex care requirements (yes)	6 (5)	2 (7)	99 (22)	<0.001
Decline in function (yes)	45 (39)	9 (30)	228 (51)	0.01
Surrogate (yes)	92 (80)	68 (77)	−	0.64
Clear end-of-life preferences (yes)	44 (38)	47 (53)	−	0.03
Advance directive (yes)	38 (33)	38 (43)	−	0.14
Want to be resuscitated				0.21
Yes	23 (20)	11 (13)	−	
Yes, depending on the prognosis	47 (41)	33 (38)		
No	45 (39)	44 (50)		

n=number of patients. SD=standard deviation

**Table 4:** Baseline characteristics of the two randomised patient groups

	Intervention n (%) (n=57)	Control n (%) (n=58)	P values
Gender (male)	40 (70)	48 (83)	0.17
Age mean (SD)	64.74 (15)	63.88 (15)	0.76
Range (min–max)	75 (19–94)	71 (19–90)	
Frequent admissions (yes)	19 (33)	28 (48)	0.11
Difficult to control symptoms (yes)	48 (84)	48 (83)	0.83
Complex care requirements (yes)	4 (7)	2 (3)	0.44
Decline in function (yes)	23 (40)	22 (38)	0.79
Surrogate (yes)	43 (75)	49 (85)	0.23
Clear end-of-life preferences (yes)	24 (42)	20 (35)	0.52
Advance directive (yes)	20 (35)	18 (31)	0.64
Want to be resuscitated			0.34
Yes	11 (19)	12 (21)	
Yes, depending on the prognosis	20 (35)	27 (47)	
No	26 (46)	19 (33)	
Ward			1.00
Dermatology	5 (9)	5 (9)	
Internal medicine	5 (9)	7 (12)	
Nephrology	3 (5)	3 (5)	
Neurology	5 (9)	5 (9)	
Oncology	16 (28)	16 (28)	
Radio-oncology	17 (30)	18 (31)	
Haematology	6 (11)	4 (7)	
Religious affiliation (yes)	44 (77)	46 (79)	0.96
Main diagnosis			0.43
Cancer	51 (90)	49 (85)	
Other	6 (11)	9 (16)	
Highest education			0.54
Lower education	3 (5)	1 (2)	
Upper education	29 (51)	33 (57)	
Higher education	25 (44)	24 (41)	
Citizenship			0.49
Swiss	48 (84)	52 (90)	
EU	5 (9)	5 (9)	
Other	4 (7)	1 (2)	
Civil status			0.28
In a relationship	39 (68)	36 (62)	
Single	5 (9)	11 (19)	
Separated	13 (23)	11 (19)	

n=number of patients. SD=standard deviation

### Primary outcome measure: end-of-life wishes known, documented and fulfilled

We report valid percentages and multiple imputation p values for the primary outcomes of resuscitation wish and last place of care in the article (table 5). Data on other end-of-life wishes and multiple imputation data are included in online supplementary file 2.

**Table 5:** Primary outcomes of patients’ wishes known and fulfilled 6 months after discharge/intervention or after death

		Intervention n (%)	Control n (%)	P values	Missings (intervention/control)	MI P values
Resuscitation	Do you want to be resuscitated?			0.014	32/21	0.037
	Yes	6 (24)	13 (35)			
	No	18 (72)	13 (35)			
	Leave decision to surrogate or physician	1 (4)	5 (14)			
	Undecided	0 (0)	6 (16)			
	Congruency between patient, surrogate and physician			0.006	23/12	0.008
	Present	21 (62)	14 (30)			
	Absent	13 (38)	32 (70)			
	Wish documented			0.021	22/13	0.041
	Yes	31 (89)	29 (64)			
	No	4 (11)	16 (36)			
	Wish fulfilled			0.821	12/2	1.000
	Yes	6 (13)	5 (9)			
	No	1 (2)	2 (4)			
	Unclear/not applicable	38 (84)	49 (88)			
Last place of care	Preferred last place of care?			0.824	32/21	0.994
	At home	17 (68)	20 (54)			
	Nursing home	2 (8)	4 (11)			
	Hospice	1 (4)	1 (3)			
	Hospital	4 (16)	7 (19)			
	Intensive care unit	0 (0)	0 (0)			
	Unsure	0 (0)	2 (5)			
	Don’t know	1 (4)	3 (8)			
	Congruency between patient, surrogate and physician			0.059	23/12	0.039
	Present	15 (44)	11 (24)			
	Absent	19 (56)	35 (76)			
	Wish documented			0.001	22/13	0.002
	Yes	17 (49)	6 (13)			
	No	18 (51)	39 (87)			
	Wish fulfilled			0.045	12/2	0.079
	Yes	13 (29)	6 (11)			
	No	7 (16)	7 (13)			
	Unclear / not applicable	25 (56)	43 (77)			
Hospitalisation	Were you hospitalised in the past six months?			0.295	22/14	0.446
	Yes	19 (54)	29 (66)			
	No	16 (46)	15 (34)			
Mortality	When did the patient die?			0.301	12/2	0.837
	Within 6 months after inclusion	19 (42)	16 (29)			
	After 6 months after inclusion	11 (24)	14 (25)			
	Alive/unclear	15 (33)	26 (46)			

n=number of patients.

MI, multiple imputation.

Six months after discharge or intervention, most concrete medical end-of-life wishes including last place of care were significantly better known to surrogates and attending physicians (triad congruency) compared with the control group. All wishes (with the exception of intravenous fluids) were more often correctly documented in medical records of patients who had received the ACP intervention compared with controls (see data on CIs and p values in online supplementary file 2). ACP significantly influenced the main outcome. At baseline, most patients in both groups wanted to be resuscitated, and a high proportion of patients wanted to link their resuscitation decisions to the likely outcome of resuscitation of which they were not aware (table 4). Six months after discharge or intervention, among patients who were able to participate in a follow-up interview, no intervention patient was undecided, and only one wanted to leave the decision to others compared with 29% of controls (p=0.014). Most intervention patients did not want to be resuscitated. More wishes of intervention patients were known (p=0.006) and documented (p=0.021). There was no statistical difference between groups regarding resuscitation wish fulfilment. Most patients in both groups wished to die at home. For 53 patients, the wish of last place of care fulfilment could be monitored, which was more often correctly documented in the medical chart (p=0.001), and was fulfilled during follow-up (p=0.045) in the intervention compared with control group. Regarding fulfilment of other end-of-life wishes (online supplementary file 2), hospitalisation and mortality (table 5), there was no statistical differences between groups.

### Secondary outcomes

#### Advance directives and surrogacy

At baseline, about one-third of patients had an AD and three quarters had an appointed surrogate decision-maker in both groups (table 4). At discharge, most intervention patients reported having an AD and an appointed surrogate decision-maker compared with 33% having an AD (p<0.001) and 82% having an appointed surrogate decision-maker (p=0.1) in the control group (table 6).

**Table 6:** Secondary outcomes: advance directive, surrogacy, decisional conflict, depression, anxiety and impact of death on surrogate decision-maker

			Intervention Mean (SD)	Control Mean (SD)	P values	Missings (intervention/control)	MI P values
Patient discharge/intervention	HADS anxiety	Mean (SD)	4.22 (3.87)	4.44 (3.25)	0.770	20/4	0.820
		Score≥8 n (%)	n=11 (30)	n=11 (20)	0.310	20/4	0.853
	HADS depression	Mean (SD)	5.73 (4.24)	5.04 (3.67)	0.42	20/4	0.328
		Score≥8 n (%)	n=12 (32)	n=15 (28)	0.630	20/4	0.903
	Decisional conflict	Mean (SD)	13.47 (15.08)	36.28 (24.44)	<0.001	20/7	0.000
	Advance directives	Yes n (%)	34 (92)	18 (33)	<0.001	20/4	0.000
	Surrogate	Yes n (%)	35 (95)	44 (82)	0.100	20/4	0.256
Patient 6 months after discharge/intervention	HADS anxiety	Mean (SD)	3.72 (2.79)	3.9 (3.73)	0.83	32/19	0.712
		Score≥8 n (%)	n=3 (12)	n=8 (21)	0.353	32/19	0.114
	HADS depression	Mean (SD)	4.68 (3.36)	4.41 (3.44)	0.76	32/19	0.721
		Score≥8 n (%)	n=5 (20)	n=7 (18)	0.839	32/19	0.602
	Decisional conflict	Mean (SD)	14.44 (13.10)	33.51 (23.99)	<0.001	32/20	0.000
	Advance directives	Yes n (%)	27 (100)	17 (44)	0.004	32/19	0.001
	Surrogate	Yes n (%)	27 (100)	30 (77)	0.040	32/19	0.009
Surrogate 6 months after discharge/intervention or 3 months after death	HADS anxiety	Mean (SD)	6.11 (5.20)	6.35 (3.41)	0.80	19/6	0.748
		Score≥8 n (%)	n=15 (40)	n=19 (37)	0.777	19/6	0.828
	HADS depression	Mean (SD)	5.45 (5.74)	5.37 (4.32)	0.94	19/6	0.889
		Score≥8 n (%)	n=10 (26)	n=10 (19)	0.431	19/6	0.543
	Decisional conflict	Mean (SD)	20.18 (14.96)	40.36 (23.42)	<0.001	33/23	0.000
	Impact of event	Mean (SD)	44.15 (15.04)	47.56 (12.90)	0.52	44/42	0.034
		Score≥33 n (%)	n=10 (77)	n=15 (94)	0.260	44/42	0.340

n=number of patients.

HADS, Hospital Anxiety and Depression Scale; MI, multiple imputation.

Six months after discharge, all patients interviewed in the intervention group had an appointed surrogate decision-maker and an AD, compared with controls where 77% had an appointed surrogate (p=0.04) and 44% had an AD (p=0.004).

#### Decisional conflict, stress, anxiety and depression

Decisional conflict in patients and surrogates regarding medical treatment in future emergency situations was significantly lower in the intervention compared with control groups at discharge (p<0.001, patients only) and 6 months after discharge or intervention (patients and surrogates p<0.001) (table 6). Anxiety and depression scores were higher in surrogates than in patients in both groups with no statistical difference between groups. Three months after death, the impact of the event on surrogates was extremely high in both groups, with scores >33, indicating post-traumatic stress syndrome, which warranted treatment in 77% of surrogates in the intervention group compared with 94% in the control group. The mean difference reached statistical significance for a lower impact of the event in the intervention group in multiple imputation data sets (MI p=0.03) (table 6).

## Discussion

Medical services around the world aim to deliver high-quality patient-centred care at the end of life, including support for surviving relatives and to deliver this high-quality care in the place where the patient wants to be cared for until death. Worldwide, including Switzerland, most patients prefer to die at home.^15 34^ Yet, most patients in most countries die in an institution (eg, hospital or nursing home).^35^ ACP was developed as a tool to bridge the gap between the ‘is’ and the ‘ought’, a goal not properly addressed by ADs completed by patients on their own.^3–7 10^ Most trials evaluating facilitation of ACP have assessed the impact on care of elderly inpatients or patients in nursing homes^3–7 10^ and no trial has combined ACP facilitation with evidence-based decision aids. The ongoing ACTION study^36^ is testing the effect of a Respecting Choices-based ACP facilitation on hospitalised patients with cancer in a multinational, multicentre cluster-randomised trial in six countries. This study promises to deliver important insights on the impact of highly skilled ACP facilitation in this patients group. Different from our trial, quality of life and symptom burden 2.5 months post intervention is the primary outcome and decision aids are not included in the intervention. Our pragmatic randomised controlled trial is therefore unique in investigating whether a newly introduced ACP facilitation strategy using decision aids could further improve patient-centred care and in measuring concordance of caregiver knowledge and fulfilment of treatment and care preferences in severely ill medical inpatients, predominantly suffering from cancer.

### Feasibility of ACP for severely ill patients in an acute hospital setting

As indicated in table 3, although we screened 1464 patients, due to the study protocol, we randomised only 115 patient–surrogate dyads. Importantly, ACP can support surrogates of patients not fully capable of decision-making, patients not able to name a surrogate or a responsible physician or patients speaking different languages. However, these groups are under-represented in most ACP studies including ours. We obtained some information on the characteristics of excluded patients and non-participants through capturing full screening results and the non-participation questionnaire, but cannot exclude non-response bias. Our screening results show that introducing ACP to patients with a positive 12 months surprise question in an acute hospital setting may be too late or not feasible. If hospital stays are too short without scheduled follow-up, or patients are too sick, ACP consultations may be impeded. Unfortunately, ACP is not routinely initiated in the ambulatory setting by general practitioners or specialists even in countries with a highly developed palliative care culture.^37^ It is therefore important that severely ill patients be exposed to ACP in all possible places of care, including the acute hospital setting, given our current findings which are consistent with those of an Australian trial on elderly medical inpatients, aged>80.^6^

### One size does (not always) fit all: differences between patient groups

Compared with similar studies, our refusal rate was high, mostly triggered by surrogates, who stated that they felt too stressed. In the Australian study,^6^ 0% of the intervention and 15% of control group surrogates indicated a post-traumatic stress syndrome on the impact of event scale after death while in our study we found the rates to be 77% and 94%, respectively (table 6, scores of the impact of event scale≥33). The high level of surrogate stress and depression in younger patients, suffering from oncological disease, is known.^38^ Focusing on general goals of care in ACP is very helpful.^4–6^ Yet, most of our intervention patients used explicit evidence-based information provided in decision aids to reach decisions on specific treatments (such as resuscitation or tube feeding) and expressed concrete end-of-life wishes in concordance with their general goals of care. We therefore suggest that, compared with elderly patients, younger severely -ill patients may have a greater need to express concrete wishes regarding specific treatments, which can be facilitated through the use of focused decision aids.

### Strengths and limitations

The main strength of our study is the double-blind randomised controlled pragmatic study design to determine the feasibility and impact of introducing an in-house ACP facilitation programme. We gained important insights regarding who may benefit, and for whom and to what extent, an ACP programme in an acute hospital setting may be too late or not feasible (table 3). Other strengths of our study are the inclusion of decision aids for more specific and informed decision-making, the length of follow-up, the inclusion of surrogates, physicians and medical record data and their triangulation, allowing construction of a tool to assess concordance of patients wishes and concrete wish fulfilment. Our sample is clearly skewed towards male patients with cancer; however, we did not find any evidence in the literature on gender preferences impacting ACP and therefore expect our findings to be generalisable to both genders. Our observation that surrogates were not only extremely stressed, but also very influential regarding the decision to participate, with female surrogates being more positive towards participation (which might explain the gender bias), remains anecdotal but deserves further study. Our initial screening based on the surprise question answered by physicians likely led to the predominance of patients with cancer being included in the study as the prognosis is generally easier to anticipate in this group. Other studies on illness trajectories have also discussed the challenges of ACP and prognostication in internal medicine conditions other than cancer.^39 40^ Our study therefore under-represents the potential benefit of ACP for general internal medicine patients in an acute hospital setting. In general, the study was underpowered for the effective evaluation of the impact of ACP on fulfilment of patient’s wishes, and we were not able to recruit the number of patients needed according to our power calculating. The significant differences that were found therefore may underestimate the true impact of ACP. In addition, we cannot rule out some selection bias as 91 patients meeting inclusion criteria were excluded by ward physicians who made their own assessment of eligibility or openness of their patients towards conversations on future care (figure 1).

Complete blinding in a complex intervention is much more difficult compared with pharmaceutical trials using placebo pills. To maximally ensure blinding we delivered a ‘placebo conversation’ to patients, informed patients, physicians and surrogates that we were testing the effect of two sorts of in-hospital conversations on post-discharge care, and assessed the endpoint of concrete end-of-life wishes within 6 months after discharge to prevent an influence of the questions addressing ACP on study participants, including physicians. The trial benefited from the fact that legal requirements for ADs were only recently introduced and ACP did not exist in Switzerland at the time the trial recruitment began, making it less likely that study participants would deduce the study goal. Yet, we cannot be fully sure that the aim of the study and the assignment of patients to the intervention group was not understood by study participants, especially participating physicians. Further, we could not establish full blinding of observers since members of the study team screened and interviewed patients, surrogates, physicians and medical records. Outcome evaluation in medical records and congruency coding (tables 1 and 2) was however monitored by a blinded study member to minimise the effect of the observers being unblinded.

Due to the need to preserve blinding during the study period, we could not introduce a (potentially more beneficial^41^) regional approach, disseminate information on ACP and deliver continuous medical education to general practitioners, emergency physicians and specialised nurses in the ambulatory setting which may have reduced the positive impact of ACP on patients’ end-of-life care. A further limitation of the study is the number of missing values (tables 5 and 6) due to the endpoints being measured 6 months after discharge in a very sick ambulatory population, among whom many were too burdened to answer or who had already died, whose surrogates sometimes felt too stressed for an interview, were lost to follow-up, had incomplete data in their medical charts or their attending physician did not want to cooperate with the study team. Through our study design of combining data on patients wishes with data from surrogates, physicians and the medical chart and using multiple imputation of all patients included, we tried to minimise the effect of missing not-at-random data and obtained endpoint data in all included patients who did not withdraw their informed consent (figure 1).

## Conclusion

Introducing a 2-day ACP educational programme, followed by continuous coaching based on Respecting Patient Choices and beizeiten begleiten for non-physician hospital staff, and offering ACP consultation during hospital stay or at follow-up in regular ambulatory consultations, is feasible for severely ill adult patients with acute difficult-to-control symptoms and minor complex care requirements. One to three facilitation sessions with trained ACP facilitators helped reduce decisional conflict for emergencies and assisted patients and surrogates to make informed choices on future care. In-house ACP facilitation also increased the knowledge of attending physicians of patients’ wishes, and the documentation of wishes in medical records, slightly reduced the impact of the death of a loved one on surrogates and increased the wish fulfilment of patients regarding last place of care. Future studies should focus on concrete wish fulfilment (rather than only wishes known), the effect of ACP on broader internal medicine and surgical patients, patients who cannot make decisions themselves and patients without family or from different cultural backgrounds. Screening strategies should develop better possibilities to identify non-cancer patients who may be approaching their end of life. ACP programmes targeted to younger severely ill patients should not only include information on general goals-of-care but also deliver evidence-based decision aids and concrete emergency plans in order to better address the need of these patients.
