# Supplementary material for: Advance care planning for the severely ill in the hospital: a randomized trial
Source: BMJ Support Palliat Care. 2019 Jan 21;12(e3):e411–23. doi: 10.1136/bmjspcare-2017-001489 (PMC9380503; doi:10.1136/bmjspcare-2017-001489)
Supplement: Supplementary data [file bmjspcare-2017-001489supp001.pdf]

ACP facilitation training DAY 1

Timetable: 9 am – 4:30 pm

| | |
|---|---|
| 9.00 – 9.05 | **Welcome** |
| 9.05 – 9.25 | **Introduction of the participants including their expectations** |
| 9.25 – 9.30 | **Aims of the training program** |
| 9.30 – 9.45 | **Introduction of the ACP concept** |
| 9.45 – 10.00 | **Exchange of experience with the AD the participants had to fill in:** What was easy? What kind of support would be useful |
| 10.00 – 10.20 | **Good Beginning**/ How to start a conversation |
| 10.20 – 10.50 | **Pairs: Starting a conversation Situations:**<br>1. Patient just returned from an intervention, is tired.<br>2. Patient awakening from his nap<br>3. Patient had visitors and doesn't want to talk<br>**Exchange of experience** |
| 10.50 – 11.05 | **Break** |
| 11.05 – 11.20 | **Insecurity and stress of the counsellor during a session** |
| 11.20 – 12.00 | **How to name a substitute decision maker?** |
| 12.00 – 12.30 | **Exchange of experiences: daily practice until now plenum** |
| 12.30 - 13.15 | **Lunch Break** |
| 13.15 – 13.40 | **Discussion of values-** Role play by trainers |
| 13.40 – 14.00 | **Legal background of ACP** |
| 14.00 – 14.15 | **General Goals of Care-** Video |
| 14.15 – 14.30 | **Break** |
| 14.30 – 15.00 | **Emergency form (ÄNO/POLST) and how to use it** |
| 15.00 - 16.00 | **Exercise in pairs: Fill in an emergency form (ÄNO/POLST) of the role play patient based on his/her General Goals of Care:** What would he/she have wanted? |
| 16.00 – 16.10 | **Information concerning homework: Fill in a complete AD with a friend or relative** |
| 16.10 – 16.30 | **Open questions and good bye** |

ACP facilitation training DAY2

Timetable: 9am – 4pm

| | |
|---|---|
| 9.00 – 9.05 | **Welcome** |
| 9.05 – 9.55 | **Questions concerning the study plan** |
| 10.00 – 10.30 | **Experiences with homework** |
| 10.30 – 10 .45 | **Break** |
| 10.45 – 11.30 | **Interaction between General Goals of care and Decision Aids**<br>Medical background for goals of care, examples<br>Logic of the AD including emergency forms |
| 11.30 – 11.50 | **Excercise:** How to recognize inconsistant/invalid ADs (betwee values, goals of care, medical measures) |
| 11.50 – 12 .30 | **Discussion** |
| 12.30 – 13.15 | **Lunch break** |
| 13.15 – 13.30 | **Introduction of the following exercise** |
| 13.30 – 14.30 | **Role play (pairs with a observer)** |

| | |
|---|---|
| | Fill in an AD<br>1. Medical situation: COPD patient<br>2. Relationship conflict, surrogate afraid to take responsibility<br>3. No medical indication for CPR but patient wants «ALL» |
| 14.30 – 15.00 | **Exchange of experiences** |
| 15.00 – 15.15 | **Break** |
| 15.30 – 15. 45 | **Information about the training sessions with the actors** |
| 15.30 – 15. 45 | **Open questions** |
| 15. 45 – 16.00 | **Evaluation and good-bye** |

Follow-up: Individual coaching support of facilitators by the MAPS study team ACP trainers up to 10 hours; ACP facilitation meetings every two months to exchange experiences
