# Supplementary material for: Advance care planning for the severely ill in the hospital: a randomized trial
Source: BMJ Support Palliat Care. 2019 Jan 21;12(e3):e411–23. doi: 10.1136/bmjspcare-2017-001489 (PMC9380503; doi:10.1136/bmjspcare-2017-001489)
Supplement: Supplementary data [file bmjspcare-2017-001489supp002.pdf]

Appendix: Supplementary file

Table of contents

Appendix: Supplementary file 1
1. Results 2
1.1. Tables 2
1.1.1. Table 1. Primary outcomes with multiple imputation (MI) 2
1.1.2. Table 2. Secondary outcome with multiple imputation (MI) 3
1.1.3. Table 3. Dyad congruency with multiple imputation (MI) 4
1.2. Graphs 6
1.2.1. Antibiotics 6
1.2.2. Artificial nutrition 6
1.2.3. Dialysis 8
1.2.4. Intravenous fluids 9
1.2.5. Intubation 10
1.2.6. Last place of care 11
1.2.7. Resuscitation 12
1.2.8. Sedation 13
1.2.9. Residual analysis 14
2. Statistical methods 15
2.1. Categorical variables: Why not using Fisher-exact test? 15
2.2. Dichotomuous Variable: Why using Bayes logistic regression when there are few events? 15
2.3. Continuous variables: Why not Levene’s test 16
2.4. Pooling of Standard Errors (Rubin’s Rules) 16
2.5. Pooling of $\chi 2$-statistics 17
2.6. Multiple imputation 18

1. Results

1.1. Tables

1.1.1. Table 1. Primary outcomes with multiple imputation (MI)

	Intervention n (%)	Control n (%)	OR (CI) Difference % (CI)	P-value	Missings (Intervention / Control)	Intervention MI n (%) (n=57)	Control MI n (%) (n=58)	OR (CI) Difference % (CI)	P-value OR (CI)
**Resuscitation**									
Do you want to be resuscitated?				0.014	32/21				0.037
Yes	6 (24)	13 (35)				19 (33)	19 (32)		
No	18 (72)	13 (35)				33 (58)	21 (36)		
Leave decision to surrogate or physician	1 (4)	5 (14)				6 (10)	11 (18)		
Undecided	0 (0)	6 (16)				0 (0)	8 (13)		
Congruency between patient, surrogate and physician			3.69 (1.45,9.40)	0.006	23/12			3.25 (1.36,7.74)	0.008
Present	21 (62)	14 (30)	31 (10.27,52.39)			34 (60)	18 (32)	28 (8.25,48.30)	
Absent	13 (38)	32 (70)				24 (40)	40 (68)		
Wish documented			3.79 (1.23,11.75)	0.021	22/13			3.50 (1.05,11.67)	0.041
Yes	31 (89)	29 (64)	24 (6.61,41.64)			48 (85)	36 (62)	22 (3.33,41.67)	
No	4 (11)	16 (36)				10 (15)	22 (38)		
Wish fulfilled				0.821	12/2				1.000
Yes	6 (13)	5 (9)				7 (13)	5 (9)		
No	1 (2)	2 (4)				4 (7)	3 (5)		
Unclear / not applicable	38 (84)	49 (88)				46 (80)	50 (86)		
**Last place of care**									
Preferred last place of care?				0.824	32/21				0.994
At home	17 (68)	20 (54)				29 (51)	24 (42)		
Nursing home	2 (8)	4 (11)				13 (23)	10 (17)		
Hospice	1 (4)	1 (3)				8 (14)	7 (12)		
Hospital	4 (16)	7 (19)				6 (10)	10 (18)		
Intensive care unit	0 (0)	0 (0)				0 (1)	1 (2)		
Unsure	0 (0)	2 (5)				0 (0)	2 (4)		
Don't know	1 (4)	3 (8)				1 (2)	3 (5)		
Congruency between patient, surrogate and physician			2.51 (0.96,6.55)	0.059				2.42 (1.05,5.60)	0.039
Present	15 (44)	11 (24)	20 (-0.54,40.95)		23/12	26 (46)	15 (26)	20 (1.30,38.56)	
Absent	19 (56)	35 (76)				32 (54)	43 (74)		
Wish documented			6.14 (2.07,18.18)	0.001	22/13			4.74 (1.77,12.66)	0.002
Yes	17 (49)	6 (13)	35 (15.93,54.55)			28 (48)	10 (17)	32 (12.49,50.89)	
No	18 (51)	39 (87)				31 (52)	48 (83)		
Wish fulfilled				0.045	12/2				0.079
Yes	13 (29)	6 (11)				15 (27)	6 (10)		
No	7 (16)	7 (13)				12 (20)	8 (13)		
Unclear / not applicable	25 (56)	43 (77)				30 (53)	44 (76)		
**Intubation**									
Do you want to be intubated?				0.088	32/21				0.901
Yes	3 (12)	5 (14)				10 (17)	8 (13)		
No	15 (60)	14 (38)				26 (46)	20 (35)		
Leave decision to surrogate or physician	2 (8)	13 (35)				13 (22)	21 (37)		
Undecided	5 (20)	5 (14)				8 (15)	9 (15)		
Congruency between patient, surrogate and physician			2.59 (0.92,7.31)	0.072				2.57 (0.97,6.78)	0.057
Present	12 (35)	8 (17)	18 (-1.54,37.35)		23/12	22 (39)	12 (20)	19 (-0.15,38.04)	
Absent	22 (65)	38 (83)				36 (61)	47 (80)		
Wish documented			4.38 (1.44,13.39)	0.009	22/13			3.38 (1.30,8.84)	0.013
Yes	30 (86)	26 (58)	28 (9.43,46.45)			47 (82)	33 (57)	25 (6.48,42.88)	
No	5 (14)	19 (42)				12 (18)	25 (43)		
Wish fulfilled				1.000	12/2				0.990
Yes	2 (4)	3 (5)				2 (4)	3 (5)		
No	0 (0)	0 (0)				2 (4)	0 (1)		
Unclear / not applicable	43 (96)	53 (95)				53 (93)	55 (94)		
**Dialysis**									
Do you want to receive dialysis?				0.432	32/21				0.882
Yes	2 (8)	6 (16)				6 (11)	9 (15)		
No	10 (40)	8 (22)				22 (38)	14 (25)		
Leave decision to surrogate or physician	8 (32)	15 (41)				19 (33)	23 (39)		
Undecided	5 (20)	8 (22)				10 (17)	13 (22)		
Congruency between patient, surrogate and physician			1.78 (0.57,5.53)	0.317				1.63 (0.56,4.75)	0.368
Present	8 (24)	7 (15)	9 (-8.91,26.96)		24/12	16 (28)	11 (19)	9 (-9.71,26.86)	
Absent	25 (76)	39 (85)				42 (73)	47 (81)		
Wish documented			9.87 (2.84,34.29)	0.000	22/13			8.2 (2.50,26.89)	0.001
Yes	16 (46)	3 (7)	39 (21.01,57.09)			27 (47)	6 (10)	37 (19.40,54.62)	
No	19 (54)	42 (93)				31 (53)	52 (90)		
Wish fulfilled				0.635	12/2				1.000
Yes	1 (2)	3 (5)				1 (2)	3 (5)		
No	0 (0)	0 (0)				2 (4)	1 (1)		
Unclear / not applicable	44 (98)	53 (95)				54 (95)	54 (94)		
**Artificial feeding**									
Do you want to be artificially fed?				0.906	32/21				1.000
Yes	4 (16)	7 (19)				10 (18)	10 (18)		
No	13 (52)	17 (46)				24 (43)	24 (42)		
Leave decision to surrogate or physician	4 (16)	8 (22)				15 (26)	16 (27)		
Undecided	4 (16)	5 (14)				7 (13)	8 (13)		
Congruency between patient, surrogate and physician			2.52 (0.95,6.70)	0.064	23/12			2.81 (1.19,6.60)	0.018
Present	14 (41)	10 (22)	19 (-0.95,39.83)			27 (47)	14 (24)	23 (4.48,41.27)	
Absent	20 (59)	36 (78)				31 (53)	44 (76)		
Wish documented			5.75 (2.02,16.32)	0.001	22/13			4.41 (1.70,11.40)	0.002
Yes	18 (51)	7 (16)	36 (16.22,55.53)			30 (53)	12 (20)	32 (13.33,51.59)	
No	17 (49)	38 (84)				28 (47)	46 (80)		
Wish fulfilled				0.861	12/2				1.000
Yes	5 (11)	4 (7)				5 (10)	4 (7)		
No	0 (0)	1 (2)				4 (6)	2 (3)		
Unclear / not applicable	40 (89)	51 (91)				48 (84)	52 (90)		
**Intravenous fluids**									
Do you want to receive intravenous fluids?				0.727	32/21				1.000
Yes	12 (48)	18 (49)				22 (38)	25 (43)		
No	6 (24)	6 (16)				17 (30)	13 (22)		
Leave decision to surrogate or physician	4 (16)	10 (27)				13 (22)	15 (26)		
Undecided	3 (12)	3 (8)				5 (9)	5 (8)		
Congruency between patient, surrogate and physician			1.28 (0.51,3.23)	0.602	23/12			1.26 (0.51,3.11)	0.621
Present	13 (38)	15 (33)	6 (-15.59,26.85)			23 (40)	20 (34)	5 (-15.64,26.32)	
Absent	21 (62)	31 (67)				36 (61)	38 (66)		
Wish documented			2.09 (0.83,5.25)	0.118	22/13			1.85 (0.81,4.21)	0.146
Yes	24 (69)	23 (51)	17 (-3.75,38.67)			37 (65)	29 (51)	15 (-4.77,34.26)	
No	11 (31)	22 (49)				21 (35)	29 (50)		
Wish fulfilled				0.596	12/2				0.346
Yes	14 (31)	20 (36)				16 (28)	20 (35)		
No	1 (2)	0 (0)				5 (9)	1 (1)		
Unclear / not applicable	30 (67)	36 (64)				36 (63)	37 (64)		

(Continued) Table 1. Primary outcomes with multiple imputation (MI)

	Intervention n (%)	Control n (%)	OR (CI) Difference % (CI)	P-value	Missings (Intervention / Control)	Intervention MI n (%) (n=57)	Control MI n (%) (n=58)	OR (CI) Difference % (CI)	P-value OR (CI)
**Antibiotics**									
Do you want to receive antibiotics against pneumonia?				0.150	32/21				0.796
Yes	12 (48)	18 (49)				23 (40)	25 (43)		
No	4 (16)	4 (11)				15 (26)	12 (21)		
Leave decision to surrogate or physician	3 (12)	12 (32)				10 (18)	16 (28)		
Undecided	6 (24)	3 (8)				10 (17)	5 (8)		
Congruency between patient, surrogate and physician			2.23 (0.85,5.83)	0.103	23/12			2.16 (0.76,6.18)	0.15
Present	14 (41)	11 (24)	17 (-3.37,37.89)			24 (43)	15 (26)	17 (-6.10,40.33)	
Absent	20 (59)	35 (76)				34 (57)	43 (74)		
Wish documented			5.92 (2.22,15.81)	0.000	22/13			5.22 (2.16,12.62)	0.000
Yes	22 (63)	10 (22)	41 (20.54,60.73)			36 (62)	14 (24)	38 (19.80,56.49)	
No	13 (37)	35 (78)				23 (38)	44 (76)		
Wish fulfilled				1.000	12/2				0.918
Yes	13 (29)	16 (29)				15 (27)	16 (28)		
No	0 (0)	0 (0)				4 (7)	1 (1)		
Unclear / not applicable	32 (71)	40 (71)				38 (66)	41 (71)		
**Sedation for symptom reduction**									
Do you want to be sedated for symptom reduction?				0.278	32/21				0.501
Yes	10 (40)	14 (38)				20 (36)	19 (32)		
No	8 (32)	5 (14)				21 (36)	13 (22)		
Leave decision to surrogate or physician	5 (20)	13 (35)				12 (21)	20 (34)		
Undecided	2 (8)	5 (14)				4 (8)	7 (12)		
Congruency between patient, surrogate and physician			2.66 (0.91,7.84)	0.075	23/12			2.67 (0.95,7.48)	0.063
Present	11 (32)	7 (15)	17 (-1.71,35.98)			21 (37)	11 (18)	19 (-0.15,37.63)	
Absent	23 (68)	39 (85)				37 (63)	48 (82)		
Wish documented			3.31 (1.26,8.69)	0.015	22/13			2.85 (1.18,6.88)	0.02
Yes	17 (49)	10 (22)	26 (5.81,46.88)			28 (49)	15 (25)	24 (4.49,42.70)	
No	18 (51)	35 (78)				30 (51)	44 (75)		
Wish fulfilled				0.528	12/2				0.351
Yes	13 (29)	12 (21)				15 (26)	12 (21)		
No	0 (0)	0 (0)				5 (8)	1 (1)		
Unclear / not applicable	32 (71)	44 (79)				38 (66)	45 (78)		
**Hospitalisation**									
Were you hospitalised in the past six months?			0.61 (0.25,1.53)	0.295	22/14			0.7 (0.28,1.75)	0.446
Yes	19 (54)	29 (66)	-12 (-33.27,10.02)			31 (54)	37 (63)	-8.6 (-30.40,13.31)	
No	16 (46)	15 (34)				27 (46)	22 (37)		
**Mortality**									
When did the patient die?				0.301	12/2				0.837
Within six months after inclusion	19 (42)	16 (29)				23 (40)	17 (29)		
After six months after inclusion	11 (24)	14 (25)				16 (27)	15 (25)		
Lives/Unclear	15 (33)	26 (46)				19 (33)	27 (46)		

1.1.2. Table 2. Secondary outcome with multiple imputation (MI)

		Intervention Mean (SD)	Control Mean (SD)	OR or Difference (CI)	P-value	Missings (Intervention / Control)	Intervention MI Mean (SD) (n=57)	Control MI Mean (SD) (n=58)	OR or Difference (CI)	P-value
Patient Discharge	HADS Anxiety Mean(SD)	4.22 (3.87)	4.44 (3.25)	-0.22 (-1.77,1.32)	0.770	20/4	4.30 (3.29)	4.44 (3.18)	-0.14 (-1.34,1.06)	0.820
	Score ≥8 (%)	n=11 (30)	n=11 (20)	9.36 (-8.87,27.59)	0.310	20/4	11.6 (20.4)	11.0 (19.0)	1.39 (-13.26,16.03)	0.853
	HADS Depression Mean(SD)	5.73 (4.24)	5.04 (3.67)	0.69 (-1.02,2.40)	0.42	20/4	5.75 (3.63)	5.06 (3.58)	0.69 (-0.70,2.10)	0.328
	Score ≥8 (%)	n=12 (32)	n=15 (28)	4.65 (-14.59,23.90)	0.630	20/4	14.7 (25.8)	15.6 (26.9)	-1.11 (-18.92,16.71)	0.903
	Decisional Conflict Mean(SD)	13.47 (15.08)	36.28 (24.44)	-22.81 (-31.21,-14.40)	<0.001	20/7	13.54 (12.29)	36.37 (22.95)	-22.83 (-29.59,-16.07)	0.000
	Advance directives Yes (%)	34 (92)	18 (33)	19.22 (5.68,65.04)	<0.001	20/4	50.3 (88.2)	19.6 (33.8)	14.78 (5.43,40.20)	0.000
	Surrogate Yes (%)	35 (95)	44 (82)	3.3 (0.81,13.46)	0.100	20/4	51.2 (89.8)	47.0 (81.0)	2.1 (0.58,7.56)	0.256
Patient six months after discharge	HADS Anxiety Mean(SD)	3.72 (2.79)	3.9 (3.73)	-0.18 (-1.81,1.46)	0.83	32/19	3.77 (2.28)	3.99 (3.23)	-0.22 (-1.42,0.97)	0.712
	Score ≥8 (%)	n=3 (12)	n=8 (21)	-8.51 (-26.48,9.46)	0.353	32/19	3.1 (5.4)	8.3 (14.3)	-8.87 (-19.87,2.12)	0.114
	HADS Depression Mean(SD)	4.68 (3.36)	4.41 (3.44)	0.27 (-1.47,2.01)	0.76	32/19	4.68 (2.54)	4.47 (3.00)	0.21 (-0.94,1.36)	0.721
	Score ≥8 (%)	n=5 (20)	n=7 (18)	2.05 (-17.72,21.82)	0.839	32/19	5.6 (9.8)	7.6 (13.1)	-3.28 (-15.60,9.05)	0.602
	Decisional Conflict Mean(SD)	14.44 (13.10)	33.51 (23.99)	-19.07 (-28.46,-9.69)	<0.001	32/20	14.45 (9.02)	33.63 (19.58)	-19.18 (-24.84,-13.53)	0.000
	Advance directives Yes (%)	27 (100)	17 (44)	105.48 (4.25,2620.05)	0.004	32/19	57.0 (100.0)	25.5 (44.0)	232.22 (8.58,6287.37)	0.001
	Surrogate Yes (%)	27 (100)	30 (77)	20.97 (1.15,381.63)	0.040	32/19	57.0 (100.0)	42.8 (73.8)	55.08 (2.72,1114.58	0.009
Surrogate six months after discharge/intervention or three months after death	HADS Anxiety Mean(SD)	6.11 (5.20)	6.35 (3.41)	-0.24 (-2.18,1.69)	0.80	19/6	6.09 (4.43)	6.34 (3.30)	-0.25 (-1.73,1.24)	0.748
	Score ≥8 (%)	n=15 (40)	n=19 (37)	2.94 (-17.38,23.25)	0.777	19/6	18.7 (32.8)	20.3 (35.0)	-2.19 (-22.02,17.63)	0.828
	HADS Depression Mean(SD)	5.45 (5.74)	5.37 (4.32)	0.08 (-2.13,2.29)	0.94	19/6	5.46 (4.88)	5.34 (4.13)	0.12 (-1.58,1.83)	0.889
	Score ≥8 (%)	n=10 (26)	n=10 (19)	1.5 (0.55,4.07)	0.431	19/6	12.9 (22.6)	10.3 (17.8)	4.87 (-10.83,20.57)	0.543
	Decisional Conflict Mean(SD)	20.18 (14.96)	40.36 (23.42)	-20.18 (-30.19,-10.16)	<0.001	33/23	20.40 (10.04)	40.33 (18.34)	-19.93 (-25.42,-14.44)	0.000
	Impact of event Mean(SD)	44.15 (15.04)	47.56 (12.90)	-3.42 (-14.30,7.48)	0.52	44/42	44.27 (7.77)	47.68 (7.34)	-3.41 (-6.56,-0.26)	0.034
	Score ≥33 (%)	n=10 (77)	n=15 (94)	0.32 (0.05,2.31)	0.260	44/42	53.8 (94.4)	57.0 (98.3)	0.4 (0.06,2.62)	0.340

1.1.3. Table 3. Dyad congruency with multiple imputation (MI)

	Intervention n (%) (n=57)	Control n (%) (n=58)	OR (CI) Difference % (CI)	P-value OR (CI)	Missings (Intervention / Control)	Intervention MI n (%) (n=57)	Control MI n (%) (n=58)	OR (CI) Difference % (CI)	P-value OR (CI)
**Resuscitation**									
Congruency between patient and surrogate			8.89 (2.44,32.36)	0.001	35/23			5.82 (2.01,16.80)	0.001
Present	19 (86.4)	13 (37.1)	49.2 (27.73,70.71)			45.3 (79.5)	23.4 (40.3)	39.1 (18.32,59.93)	
Absent	3 (13.6)	22 (62.9)				12.7 (20.5)	34.6 (59.7)		
Congruency between patient and physician			4.57 (1.22,17.16)	0.024	38/36			3.15 (1.22,8.18)	0.018
Present	12 (63.2)	6 (27.3)	35.9 (7.31,64.46)			34.3 (60.2)	18.9 (32.6)	27.6 (5.98,49.20)	
Absent	7 (36.8)	16 (72.7)				23.7 (39.8)	39.1 (67.4)		
Congruency between surrogate and physician			4.16 (1.38,12.49)	0.011	30/27			2.83 (1.07,7.44)	0.035
Present	17 (63.0)	9 (29.0)	33.9 (9.70,58.16)			34.8 (61.1)	20.8 (35.9)	25.2 (2.83,47.56)	
Absent	10 (37)	22 (71)				23.2 (38.9)	37.2 (64.1)		
**Last place of care**									
Congruency between patient and surrogate			4.16 (1.27,13.65)	0.019	35/23			3.16 (1.22,8.15)	0.017
Present	18 (81.8)	17 (48.6)	42 (18.22,65.74)			42.6 (74.7)	28.2 (48.6)	29 (5.84,52.09)	
Absent	4 (18.2)	18 (51.4)				15.4 (25.3)	29.8 (51.4)		
Congruency between patient and physician			3.05 (0.80,11.60)	0.101	38/32			1.97 (0.81,4.81)	0.136
Present	8 (42.1)	5 (19.2)	22.9 (-4.00,49.75)			25.5 (44.7)	17.0 (29.3)	15.4 (-4.39,35.24)	
Absent	11 (57.9)	21 (80.8)				32.5 (55.3)	41 (70.7)		
Congruency between surrogate and physician			2.31 (0.79,6.76)	0.126	30/23			2.02 (0.84,4.88)	0.117
Present	12 (44.4)	9 (25.7)	18.7 (-4.95,42.41)			26.1 (45.8)	17.1 (29.5)	16.3 (-4.00,36.61)	
Absent	15 (55.6)	26 (74.3)				31.9 (54.2)	40.9 (70.5)		
**Intubation**									
Congruency between patient and surrogate			3 (0.98,9.14)	0.053	35/23			2.03 (0.77,5.32)	0.151
Present	12 (54.5)	10 (28.6)	26 (0.34,51.60)			29.7 (52.1)	20.3 (35.0)	17.1 (-5.89,40.10)	
Absent	10 (45.5)	25 (71.4)				28.3 (47.9)	37.7 (65)		
Congruency between patient and physician			4.44 (1.17,16.82)	0.028	38/33			2.72 (1.00,7.39)	0.049
Present	10 (52.6)	5 (20.0)	32.6 (5.25,60.02)			29.7 (52.1)	16.7 (28.8)	23.3 (1.09,45.53)	
Absent	9 (47.4)	20 (80)				28.3 (47.9)	41.3 (71.2)		
Congruency between surrogate and physician			1.91 (0.63,5.82)	0.254	30/24			1.81 (0.61,5.38)	0.288
Present	10 (37.0)	8 (23.5)	13.5 (-9.62,36.64)			23.5 (41.2)	16.3 (28.1)	13.1 (-10.86,37.11)	
Absent	17 (63)	26 (76.5)				34.5 (58.8)	41.7 (71.9)		
**Dialysis**									
Congruency between patient and surrogate			1.45 (0.48,4.41)	0.515	35/24			1.38 (0.58,3.28)	0.46
Present	9 (40.9)	11 (32.4)	8.6 (-17.32,34.43)			25.6 (44.9)	21.5 (37.1)	7.8 (-12.88,28.57)	
Absent	13 (59.1)	23 (67.6)				32.4 (55.1)	36.5 (62.9)		
Congruency between patient and physician			4.62 (1.24,17.20)	0.022	39/32			4.39 (1.61,11.93)	0.004
Present	9 (50.0)	4 (15.4)	34.6 (7.67,61.56)			30.5 (53.5)	12.2 (21.0)	32.5 (11.89,53.06)	
Absent	9 (50)	22 (84.6)				27.5 (46.5)	45.8 (79)		
Congruency between surrogate and physician			2.58 (0.73,9.18)	0.142	32/24			2.46 (0.95,6.39)	0.065
Present	7 (28.0)	4 (11.8)	16.2 (-4.43,36.90)			18.6 (32.6)	9.6 (16.6)	16.1 (-0.77,32.93)	
Absent	18 (72)	30 (88.2)				39.4 (67.4)	48.4 (83.4)		

(continued) Dyad congruency with multiple imputation (MI)

	Intervention n (%) (n=57)	Control n (%) (n=58)	OR (CI) Difference % (CI)	P-value OR (CI)	Missings (Intervention / Control)	Intervention MI n (%) (n=57)	Control MI n (%) (n=58)	OR (CI) Difference % (CI)	P-value OR (CI)
**Artificial feeding**									
Congruency between patient and surrogate			2.5 (0.82,7.60)	0.106	35/23			2.08 (0.76,5.71)	0.155
Present	11 (50.0)	10 (28.6)	21.4 (-4.27,47.13)			29.1 (51.1)	19.5 (33.6)	17.4 (-5.99,40.85)	
Absent	11 (50)	25 (71.4)				28.9 (48.9)	38.5 (66.4)		
Congruency between patient and physician			2.3 (0.63,8.39)	0.206	38/33			2.08 (0.72,5.97)	0.175
Present	8 (42.1)	6 (24.0)	18.1 (-9.70,45.91)			25.7 (45.1)	16.5 (28.4)	16.6 (-7.15,40.43)	
Absent	11 (57.9)	19 (76)				32.3 (54.9)	41.5 (71.6)		
Congruency between surrogate and physician			4.33 (1.36,13.83)	0.013	30/24			3.64 (1.32,10.06)	0.013
Present	13 (48.1)	6 (17.6)	30.5 (7.71,53.29)			28.3 (49.6)	12.5 (21.6)	28.1 (7.81,48.38)	
Absent	14 (51.9)	28 (82.4)				29.7 (50.4)	45.5 (78.4)		
**Intravenous fluids**									
Congruency between patient and surrogate			3.93 (1.26,12.28)	0.019	35/24			2.67 (0.99,7.19)	0.052
Present	15 (68.2)	12 (35.3)	32.9 (7.65,58.12)			35.2 (61.8)	22.0 (37.9)	23.8 (0.46,47.19)	
Absent	7 (31.8)	22 (64.7)				22.8 (38.2)	36 (62.1)		
Congruency between patient and physician			1.35 (0.38,4.80)	0.640	39/33			1.39 (0.60,3.26)	0.445
Present	7 (38.9)	8 (32.0)	6.9 (-22.12,35.90)			24.2 (42.5)	20.1 (34.7)	7.8 (-12.04,27.64)	
Absent	11 (61.1)	17 (68)				33.8 (57.5)	37.9 (65.3)		
Congruency between surrogate and physician			0.96 (0.33,2.76)	0.942	31/25			0.98 (0.43,2.23)	0.956
Present	10 (38.5)	13 (39.4)	-0.9 (-25.98,24.12)			23.3 (40.9)	24.0 (41.4)	-0.5 (-20.43,19.43)	
Absent	16 (61.5)	20 (60.6)				34.7 (59.1)	34 (58.6)		
**Antibiotics**									
Congruency between patient and surrogate			4.17 (1.34,13.03)	0.014	35/23			3.41 (1.14,10.18)	0.028
Present	13 (59.1)	9 (25.7)	33.4 (8.24,58.51)			33.9 (59.5)	17.6 (30.3)	29.1 (4.24,54.01)	
Absent	9 (40.9)	26 (74.3)				24.1 (40.5)	40.4 (69.7)		
Congruency between patient and physician			1.7 (0.51,5.70)	0.390	38/32			1.49 (0.59,3.73)	0.397
Present	9 (47.4)	9 (34.6)	12.8 (-16.20,41.71)			28.1 (49.3)	23.0 (39.7)	9.6 (-12.61,31.90)	
Absent	10 (52.6)	17 (65.4)				29.9 (50.7)	35 (60.3)		
Congruency between surrogate and physician			1.53 (0.55,4.30)	0.417	30/23			1.47 (0.61,3.54)	0.392
Present	12 (44.4)	12 (34.3)	10.2 (-14.31,34.62)			26.3 (46.1)	21.4 (36.9)	9.2 (-11.82,30.30)	
Absent	15 (55.6)	23 (65.7)				31.7 (53.9)	36.6 (63.1)		
**Sedation for symptom reduction**									
Congruency between patient and surrogate			2.62 (0.87,7.88)	0.087	35/23			2.09 (0.85,5.14)	0.108
Present	12 (54.5)	11 (31.4)	23.1 (-2.76,48.99)			29.8 (52.3)	20.0 (34.5)	17.8 (-3.53,39.13)	
Absent	10 (45.5)	24 (68.6)				28.2 (47.7)	38 (65.5)		
Congruency between patient and physician			4.02 (0.99,16.37)	0.052	39/32			3.35 (1.13,9.95)	0.030
Present	7 (38.9)	3 (11.5)	27.4 (1.70,53.00)			24.9 (43.7)	11.1 (19.1)	24.5 (3.01,46.09)	
Absent	11 (61.1)	23 (88.5)				33.1 (56.3)	46.9 (80.9)		
Congruency between surrogate and physician			3.57 (1.04,12.23)	0.043	31/23			2.9 (0.99,8.51)	0.053
Present	9 (34.6)	4 (11.4)	23.2 (2.08,44.29)			21.9 (38.4)	10.4 (17.9)	20.5 (0.97,40.01)	
Absent	17 (65.4)	31 (88.6)				36.1 (61.6)	47.6 (82.1)		

1.2. Graphs

1.2.1. Antibiotics

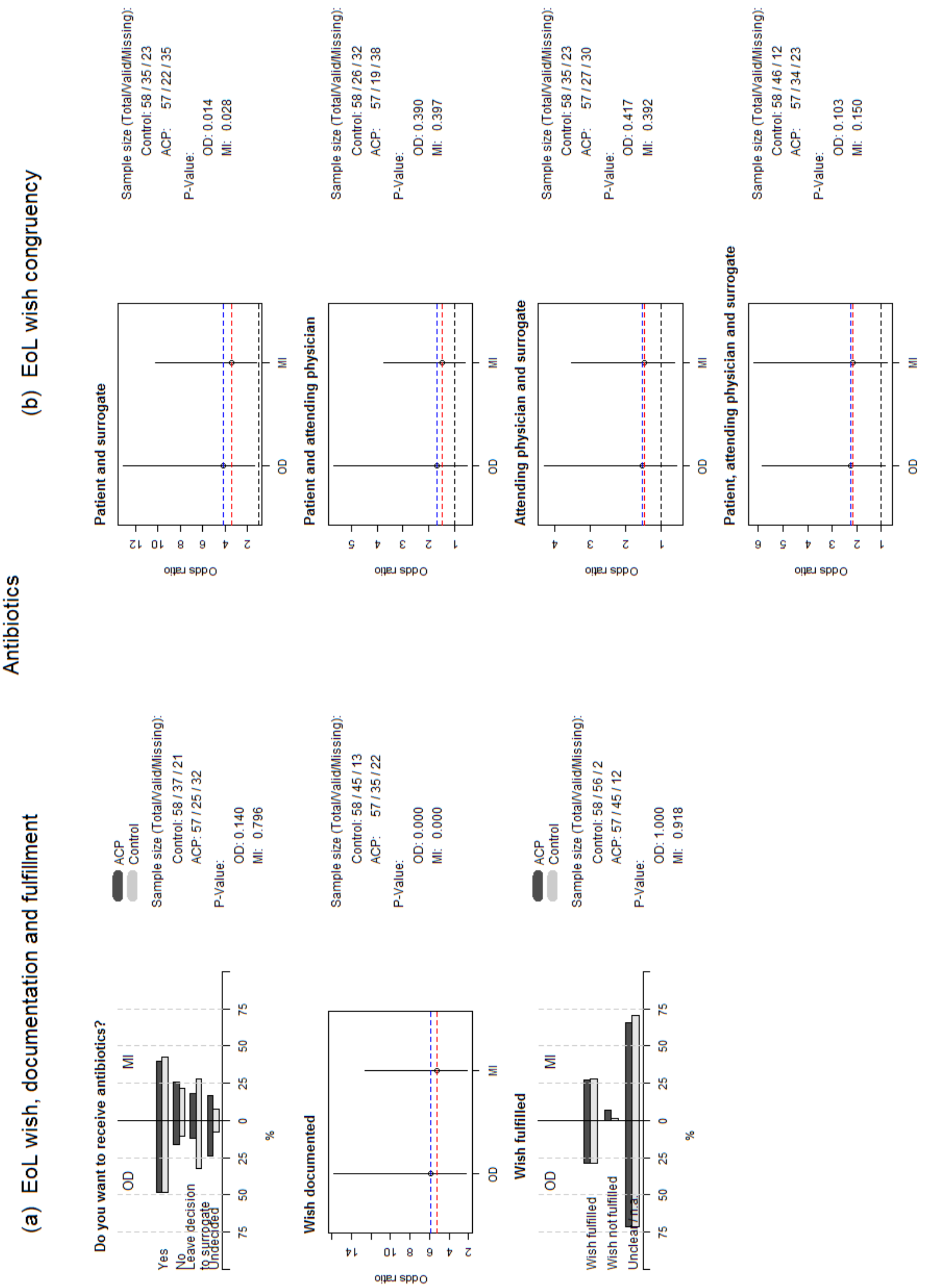

1.2.2. Artificial nutrition

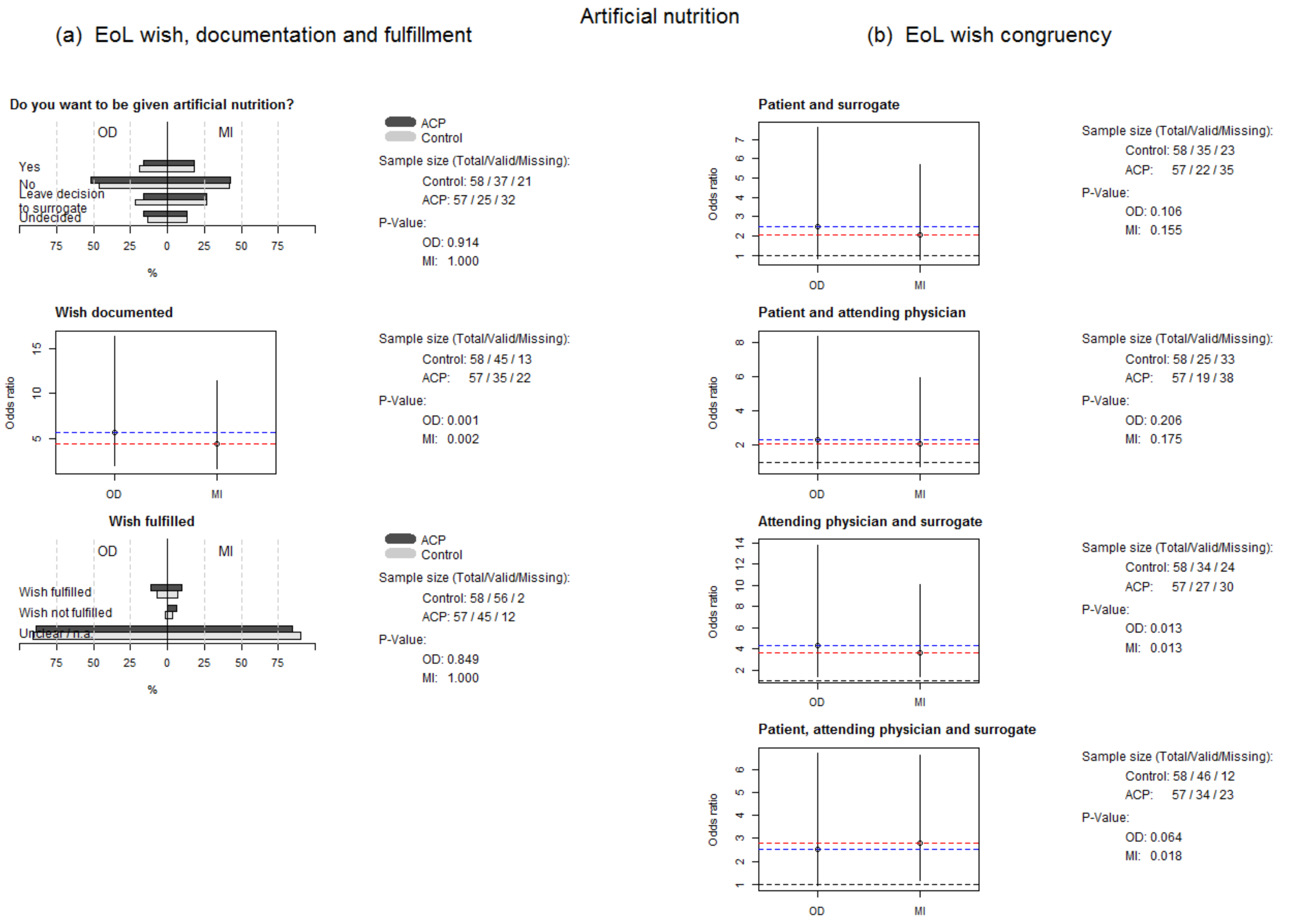
Artificial nutrition
(a) EoL wish, documentation and fulfillment
(b) EoL wish congruency
Do you want to be given artificial nutrition?
OD
MI
Yes
No
Leave decision to surrogate
Undecided
%
ACP
Control
Sample size (Total/Valid/Missing):
Control: 58 / 37 / 21
ACP: 57 / 25 / 32
P-Value:
OD: 0.914
MI: 1.000
Wish documented
Odds ratio
Sample size (Total/Valid/Missing):
Control: 58 / 45 / 13
ACP: 57 / 35 / 22
P-Value:
OD: 0.001
MI: 0.002
Wish fulfilled
Wish fulfilled
Wish not fulfilled
Unclear / n.a.
Sample size (Total/Valid/Missing):
Control: 58 / 56 / 2
ACP: 57 / 45 / 12
P-Value:
OD: 0.849
MI: 1.000
Patient and surrogate
Sample size (Total/Valid/Missing):
Control: 58 / 35 / 23
ACP: 57 / 22 / 35
P-Value:
OD: 0.106
MI: 0.155
Patient and attending physician
Sample size (Total/Valid/Missing):
Control: 58 / 25 / 33
ACP: 57 / 19 / 38
P-Value:
OD: 0.206
MI: 0.175
Attending physician and surrogate
Sample size (Total/Valid/Missing):
Control: 58 / 34 / 24
ACP: 57 / 27 / 30
P-Value:
OD: 0.013
MI: 0.013
Patient, attending physician and surrogate
Sample size (Total/Valid/Missing):
Control: 58 / 46 / 12
ACP: 57 / 34 / 23
P-Value:
OD: 0.064
MI: 0.018

1.2.3. Dialysis

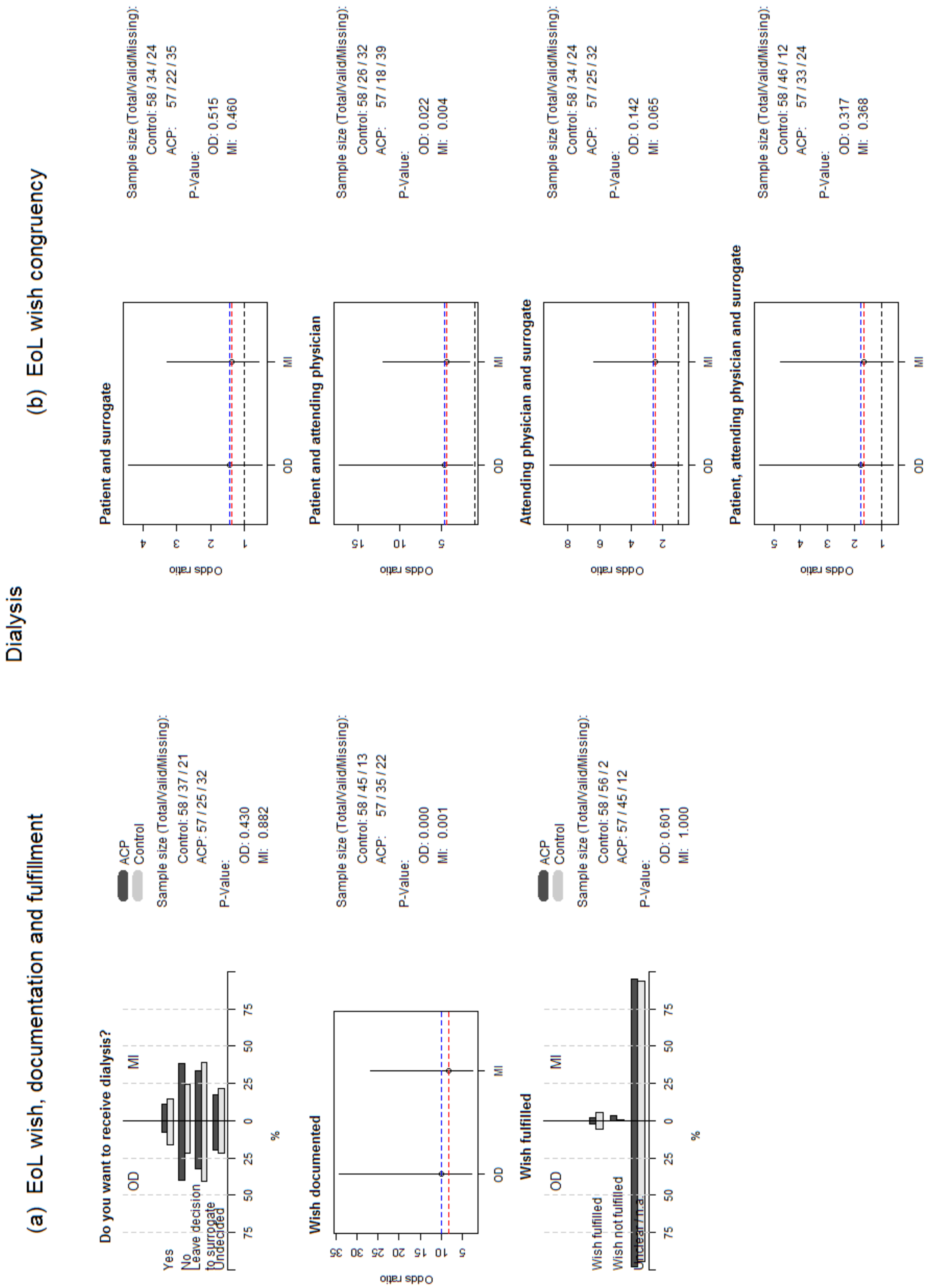

1.2.4. Intravenous fluids

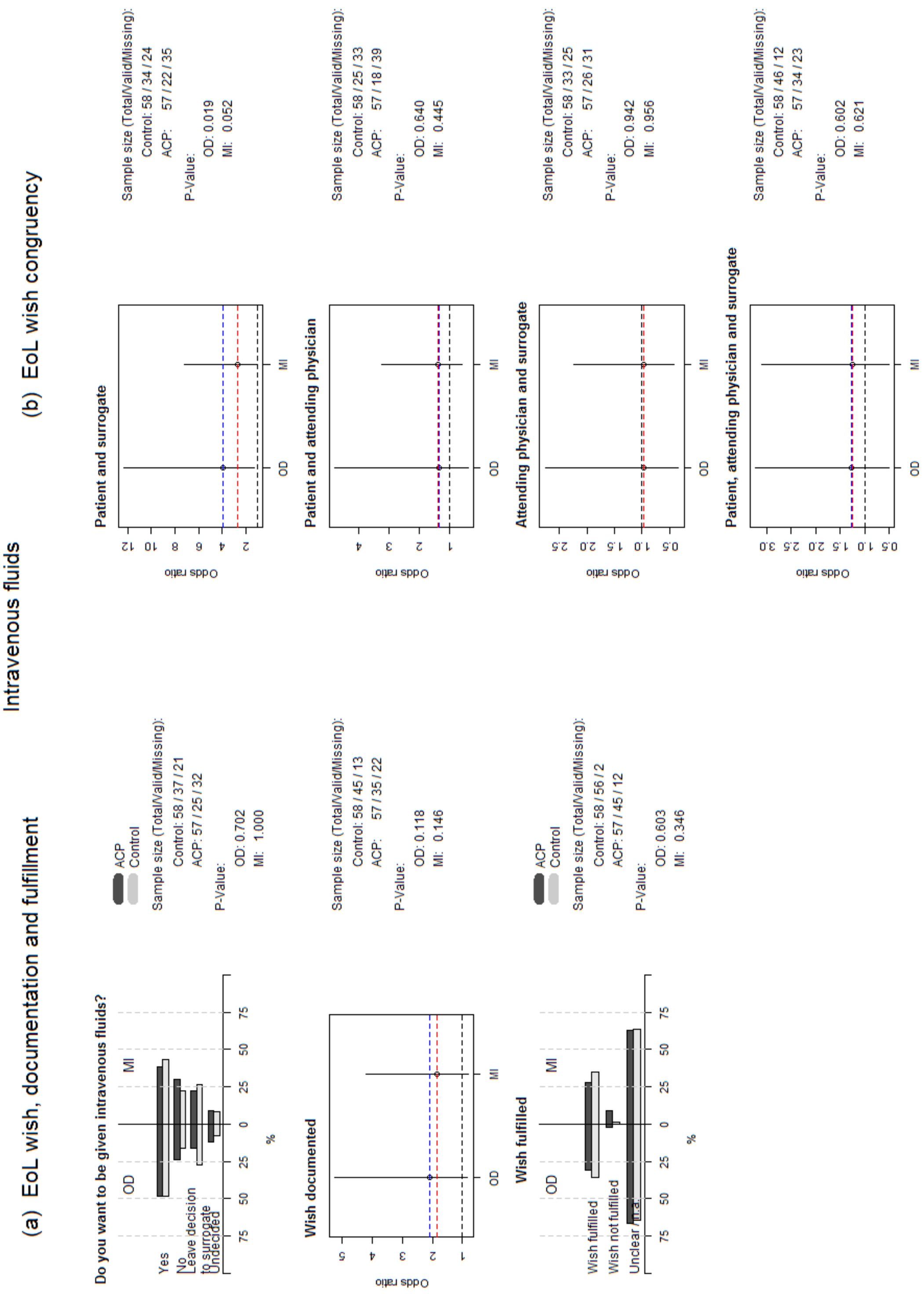

1.2.5. Intubation

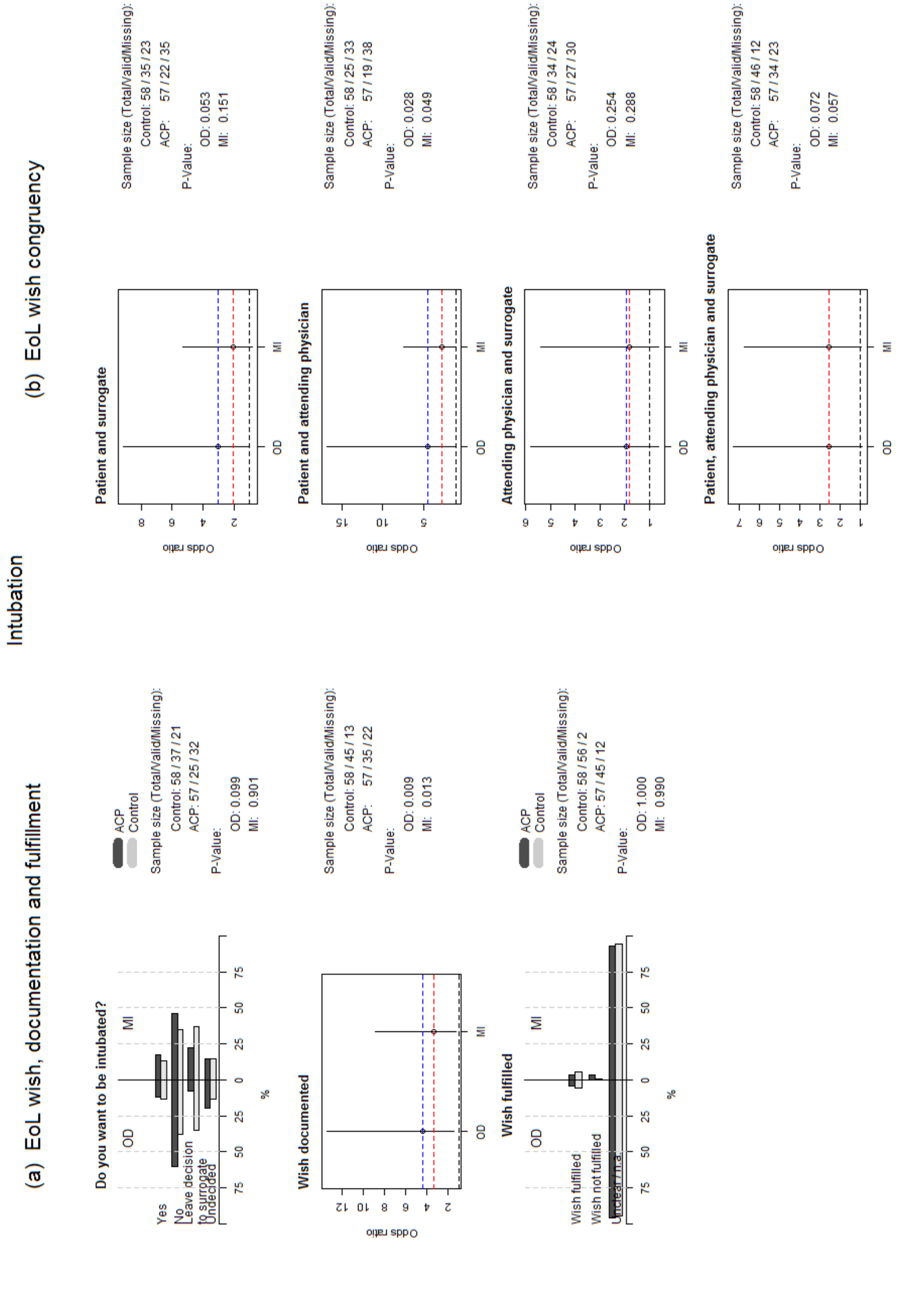

1.2.6. Last place of care

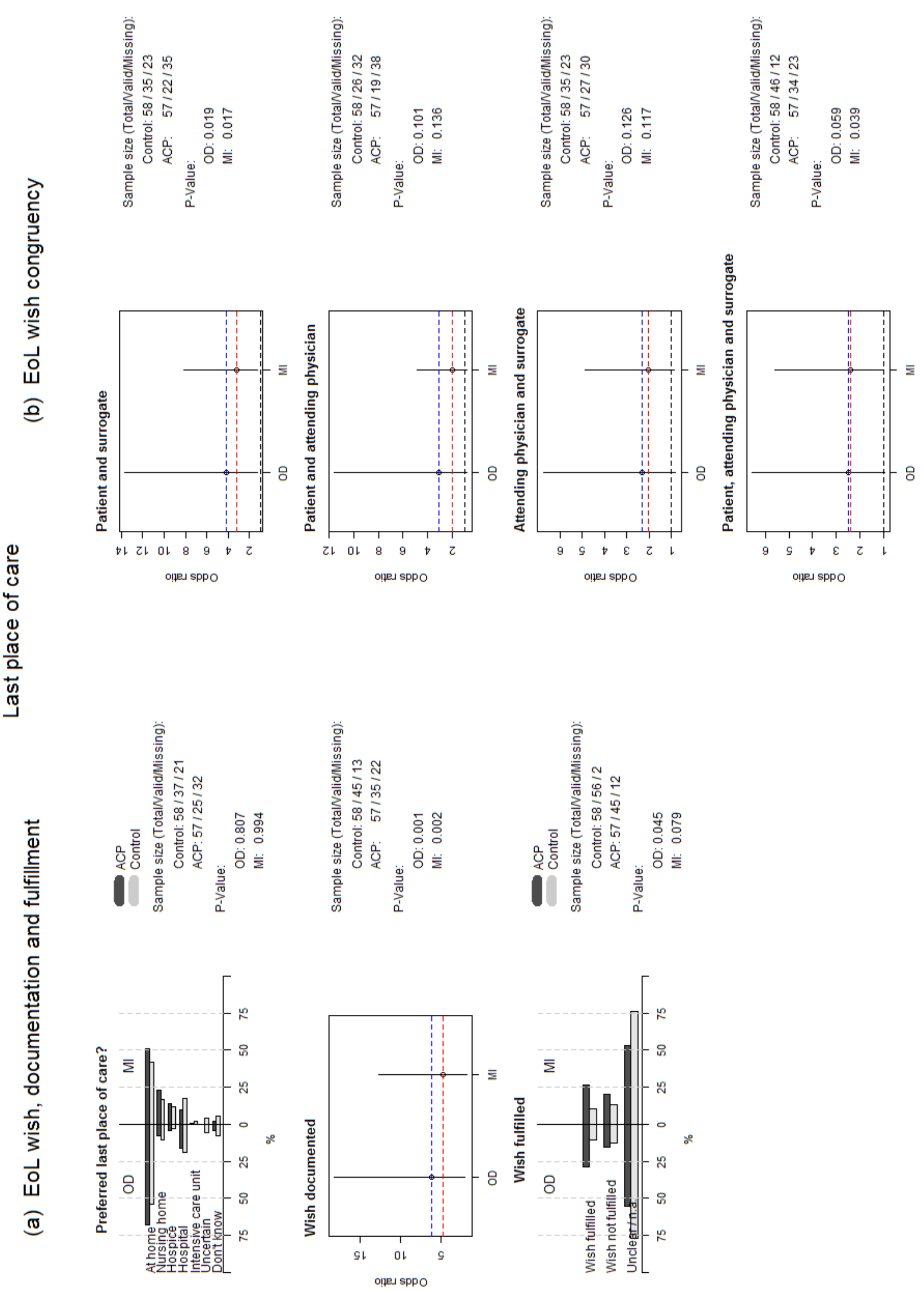

1.2.7. Resuscitation

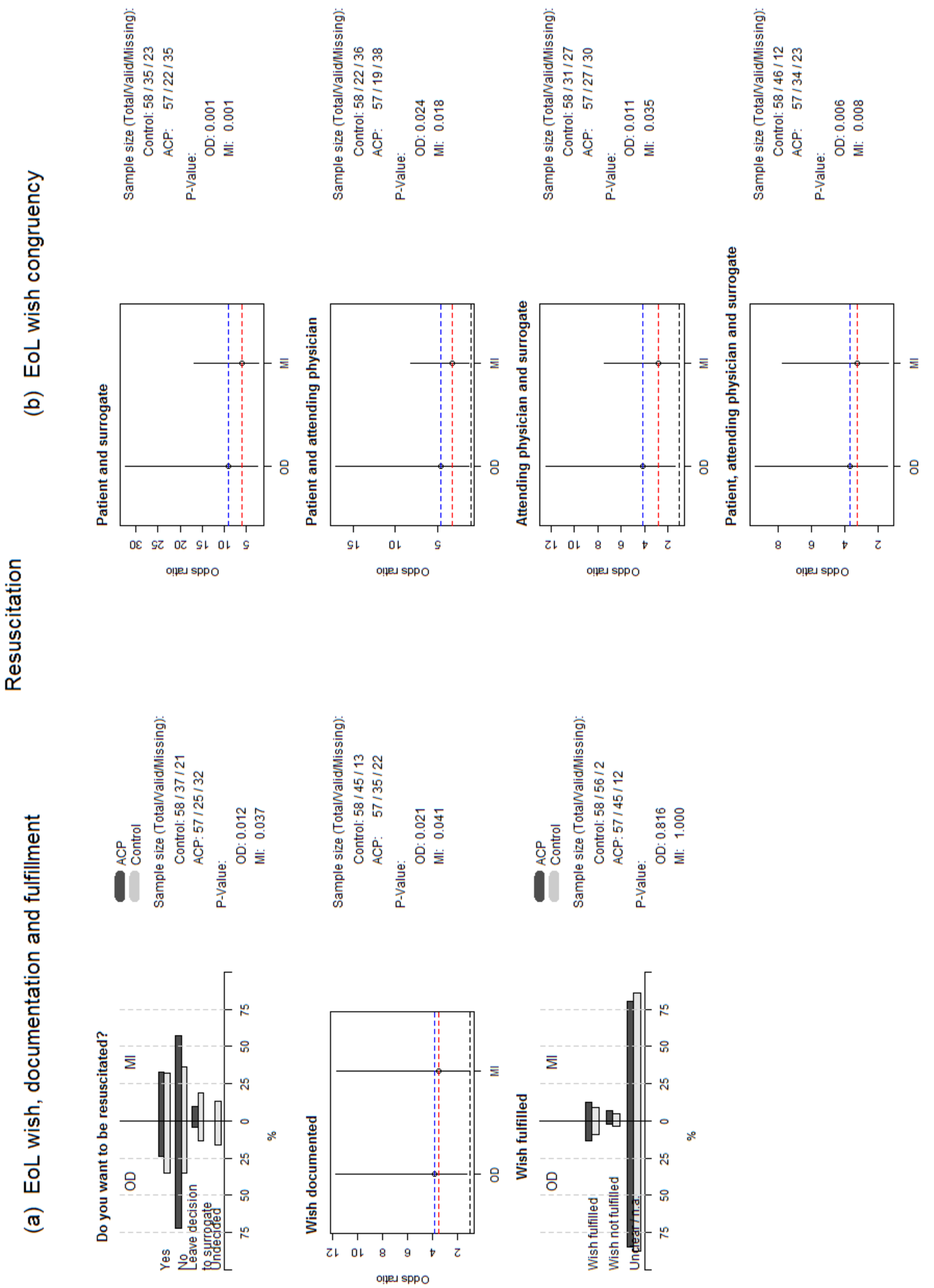

1.2.8. Sedation

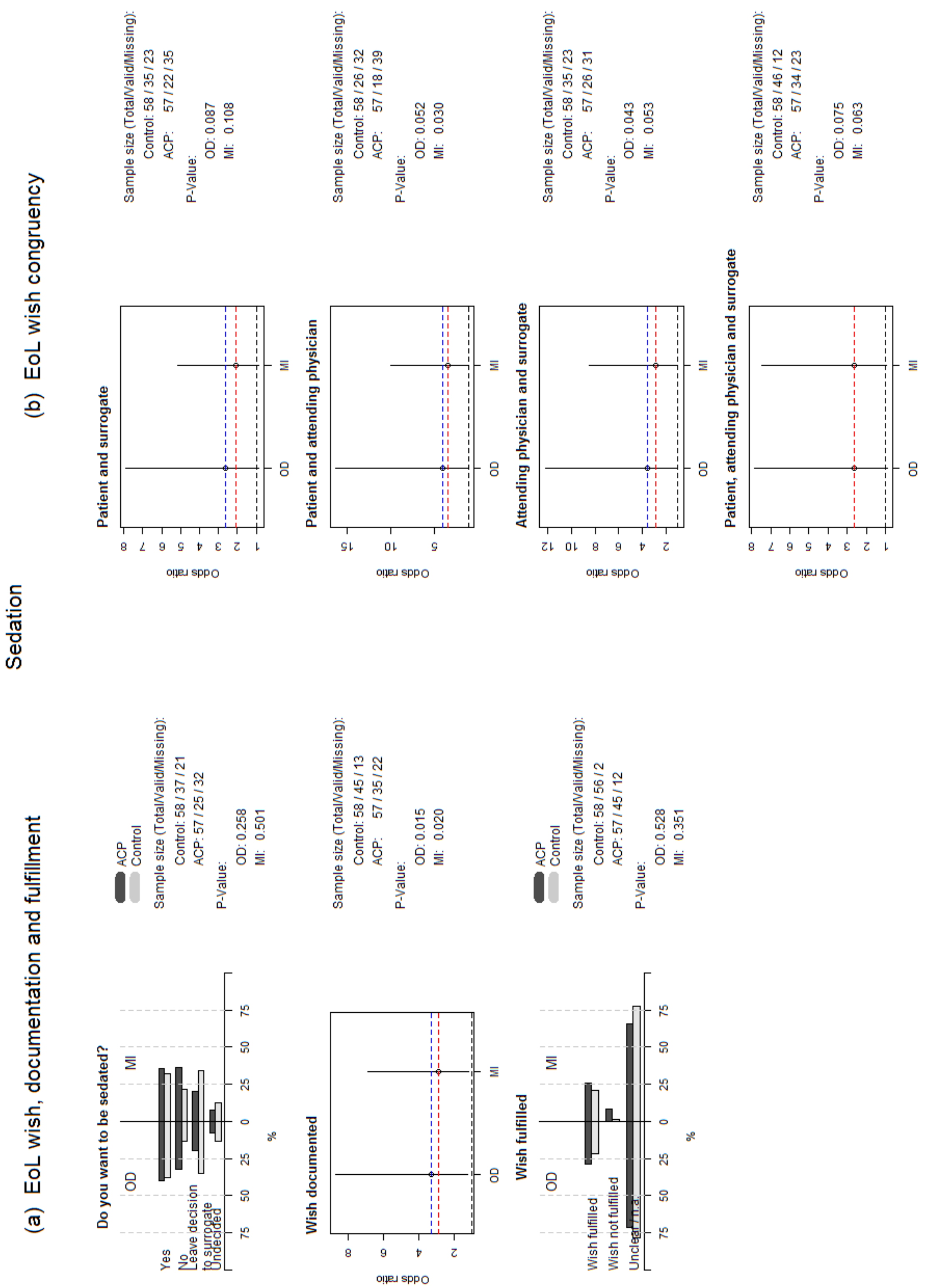

1.2.9. Residual analysis

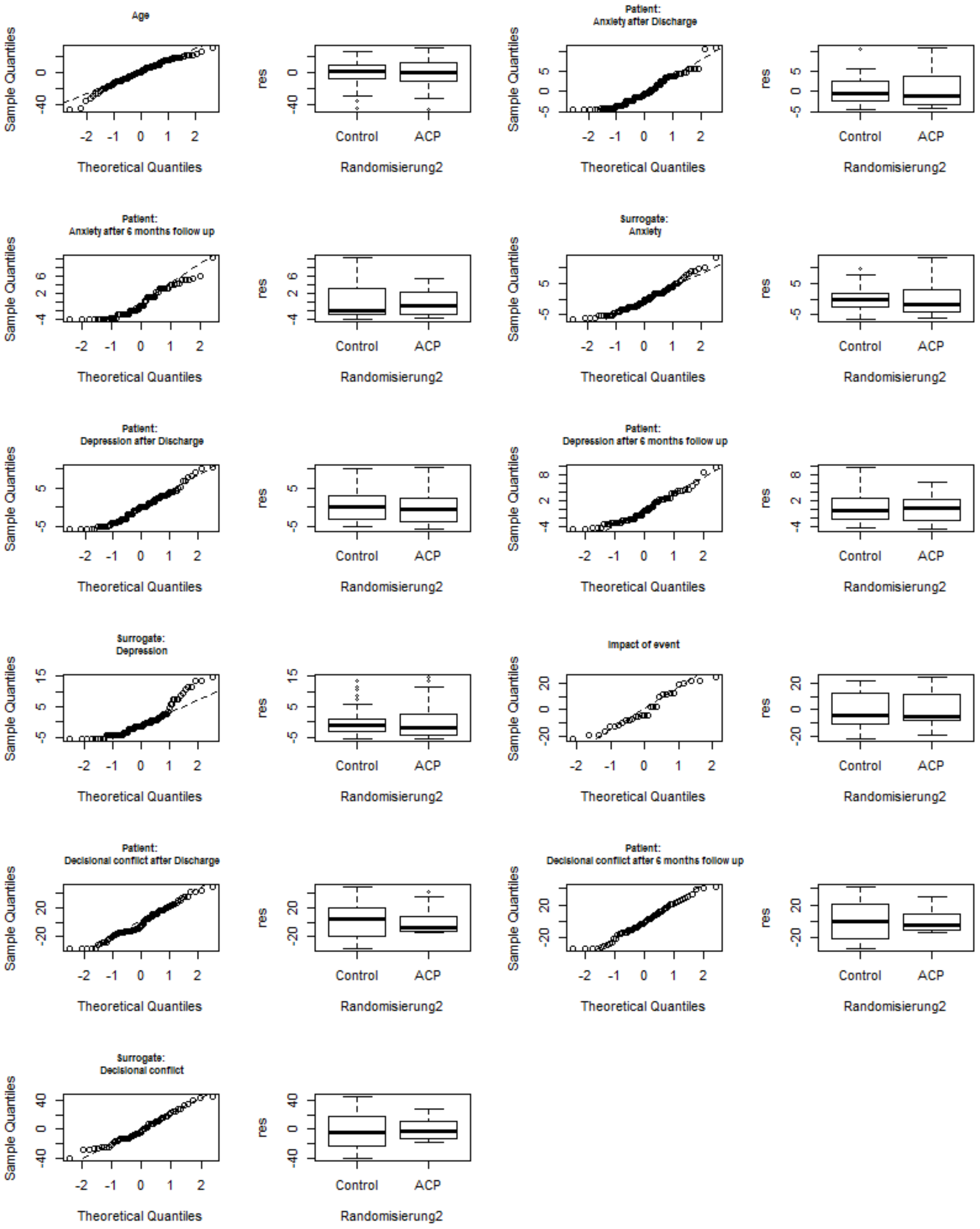

2. Statistical methods

2.1. Categorical variables: Why not using Fisher-exact test?

For the categorical variables, the chi-square test without Yates correction was used. When the expected cell frequencies was small (less than 5), a MCMC-simulation was used. Both tests were performed with R:

```
chisq.test(data)     # classical
chisq.test(data, simulate.p.value=TRUE)   # MCMC-simulation
```

Hope, A. C. A. (1968) A simplified Monte Carlo significance test procedure. J. Roy, Statist. Soc. B 30, 582–598.
Patefield, W. M. (1981) Algorithm AS159. An efficient method of generating r x c tables with given row and column totals. Applied Statistics 30, 91–97.

2.2. Dichotomuous Variable: Why using Bayes logistic regression when there are few events?

We used classical logistic regression except when there was complete separation or one cell had less than 5 observations (arbitrarily). When this was the case, we used Bayes logistic regression in R using *bayesglm* from package arm.

Justification:

Complete separation:

Andrew Gelman, Aleks Jakulin, Maria Grazia Pittau and Yu-Sung Su. (2009). "A Weakly Informative Default Prior Distribution For Logistic And Other Regression Models." The Annals of Applied Statistics 2 (4): 1360–1383.

Small number in cell:

Vittinghoff, E. and C.E. McCulloch (2006) "Relaxing the rule of ten events per variable in logistic and Cox regression." American Journal of Epidemiology 165: 710-718.

Courvoisier, D.S., C. Combescure, T. Agoritsas, A. Gayet-Ageron and T.V. Perneger (2011) "Performance of logistic regression modeling: beyond the number of events per variable, the role of data structure." Journal of Clinical Epidemiology 64: 993-1000.

2.3. Continuous variables: Why not Levene's test

To compare the mean between randomization groups we used Welch's test.

Justification:

Zimmerman, D. W. (2004), A note on preliminary tests of equality of variances. British Journal of Mathematical and Statistical Psychology, 57: 173–181. doi:10.1348/000711004849222

2.4. Pooling of Standard Errors (Rubin's Rules)

Enders, Craig K. (2010): Applied Missing Data Analysis. The Guilford Press, New York.

Page 222:

SE: Standard Errors
m: Number of imputations

Within-imputation variance V_W:

$$V_W = \frac{1}{m} \sum_{t=1}^{m} SE_t$$

Between-imputation variance V_B:

$$V_B = \frac{1}{m-1} \sum_{t=1}^{m} \left(\hat{\theta}_t - \hat{\bar{\theta}} \right)$$

Total sampling variance V_T:

$$V_T = V_W + V_{B+} \frac{V_W}{m}$$

2.5. Pooling of χ^2-statistics

van Buuren, Stef (2013): Flexible Imputation of Missing Data, Boca Raton: CRC Press.

Page: 159

Li, K.-H., Meng, X.-L., Raghunathan, T.E., and Rubin, D.B. 1991. Significance levels from repeated p-values with multiple-imputed data. Statistica Sinica, 1(1), 65-92.

1. Pool χ^2-statistics (average) $\overline{\chi^2}$:

$$\overline{\chi^2} = \frac{1}{m}\sum_{t=1}^{m}\chi_t^2$$

2. Relative increase of the variance $\overline{r_\chi}$:

$$\overline{r_\chi} = \left(1 + \frac{1}{m}\right) + \frac{1}{m} + \sum_{t=1}^{m}\left(\sqrt{\chi_t^2} - \sqrt{\overline{\chi^2}}\right)^2$$

3. Compute test-statistics D_χ:

$$D_\chi = \frac{\frac{\overline{\chi^2}}{k} - \frac{m+1}{m-1}\overline{r_\chi}}{1 + \overline{r_\chi}}$$

4. Compute Reiter's degree of freedom ν_χ:

$$\nu_\chi = k^{-\frac{3}{m}}(m-1)\left(1 + \frac{1}{\overline{r_\chi}}\right)$$

where k is degree of freedom from $\chi^2 - test$.

5. Compute p-value P_χ:

$$P_\chi = Pr\left[F_{k,\upsilon_\chi} > D_\chi\right]$$

2.6. Multiple imputation

1. According to the study design, most of the present missing data was due to the death of the participants during the study. Because of this outcome, we can assume that the missing data was missing at random (MAR), as the missing data was not dependent on the question itself, but on the inability of the respondent to answer. We can suppose that MAR might not be the case for the surrogates, as it can be argued that some specific questions from the outcome scales (ex. HADS) might not have been answered because of the reasons which are being assessed by the questions themselves (For example 25 surrogates did not answer the question "I still enjoy the things I used to enjoy"). In such cases, it could be argued that the data is missing not at random (MNAR). However, as surrogates that did not answer the scale questions, also failed to complete other questionnaires, this might imply that the data was missing not because of the questions themselves but because of other reasons. One possible reason could be the fact that their relative died (recorded by the variable Todesstatus).

2. Before performing MI, the data was split into two datasets: one with the control group and the second with the intervention group. This decision was taken to make sure that the imputed variables will be determined by the predictors exclusively within each group. "This is necessary in randomized trials, where interactions as yet unidentified between treatment and patient characteristics (covariates) may be present. If imputation is not done separately for each treatment group, estimates of interactions with treatment in the analysis model are biased toward zero." Royston P. Multiple imputation of missing values: further update of ice, with an emphasis on categorical variables. Stata J. 2009;9(3):466

To do this, the original dataset was split into two separate files (select cases function) which allowed to perform the multiple imputation in two separate files for the two randomized groups. After the multiple imputation was performed, the data was merged again.

3. Imputation specification:

a. Used system: SPSS 22

b. Number of imputations: 10

c. Variables used in the imputation procedure: Within this study, patients wishes regarding eight end of life treatment options have been assessed: resuscitation, antibiotics, dialysis, artificial nutrition, intravenous fluids, intubation, sedation and last place of care. For the multiple imputation the following variables have been included (both as a predictor and outcome for the imputation):

i. Eight variables that assess whether the patients' wishes was documented in the medical records

ii. Eight variables that assess whether the patients' wishes were fulfilled

iii. 33 variables that assess what are patients' wishes

iv. 33 variables that assess what the surrogates believe patients' wishes are

v. 33 variables that assess what the attending physicians believe patients' wishes are

vi. One variable that assesses whether the patient has any end of life wishes

vii. One variable that assesses whether the surrogates know if the patient has any end of life wishes

viii. One variable that assesses whether the attending physicians know if the patient has any end of life wishes

ix. Seven patient demographical questions: age, gender, education, religion, religiousness, civil status, main diagnosis

x. Four screening questions: presence of frequent admissions, difficult to control symptoms, complex care requirements, decline in functioning

xi. Four baseline questions: presence of surrogate, advanced care directive, wish to be resuscitated, wish for a specific medical treatment

xii. Eight discharge questions: Presence of surrogate, advanced care directives, communication with medical workers about diagnosis and future wishes and 5 satisfaction questions (involvement of patients in the decision making, involvement of surrogates in the decision making, respect of the medical workers when discussing diagnosis and treatment, offered care, given information)

xiii. Two questions six months after the discharge: presence of surrogate, advanced care directive

xiv. Three HADS Anxiety scale (only the total sum): Sum of subscale for patients answers after discharge, six months after discharge and sum of subscales for surrogates

xv. Three HADS depression scale (only the total sum): Sum of subscale for patients answers after discharge, six months after discharge and sum of subscales for surrogates

xvi. Impact of event scale (only total sum)

xvii. Two decisional conflict scale (only total sum): discharge and six months after discharge

xviii. One decisional conflict sum (total sum)

xix. Period of the study when the patient died

xx. Place of death

xxi. 33 variables that assess the congruency between the patient and the surrogate regarding the end of life treatment options

xxii. 33 variables that assess the congruency between the patient and the attending physician regarding the end of life treatment options

xxiii. 33 variables that assess the congruency between the patient, the surrogate and the attending physician regarding the end of life treatment options

d. Used statistics: see section2.

e. Because the missing pattern was arbitrary, the MCMC (Markov chain Monte Carlo) imputation method was used
