# Supplementary material for: Advance care planning for the severely ill in the hospital: a randomized trial
Source: BMJ Support Palliat Care. 2019 Jan 21;12(e3):e411–23. doi: 10.1136/bmjspcare-2017-001489 (PMC9380503; doi:10.1136/bmjspcare-2017-001489)
Supplement: Supplementary data [file bmjspcare-2017-001489supp003.pdf]

CONSORT 2010 checklist of information to include when reporting a randomised trial*

Section/Topic	Item No	Checklist item	Reported on page No
**Title and abstract**			
	1a	Identification as a randomised trial in the title	Page 1
	1b	Structured summary of trial design, methods, results, and conclusions (for specific guidance see CONSORT for abstracts)	Page 2
**Introduction**			
Background and objectives	2a	Scientific background and explanation of rationale	Page 3-4
	2b	Specific objectives or hypotheses	Page 3
**Methods**			
Trial design	3a	Description of trial design (such as parallel, factorial) including allocation ratio	Page 2,3,4
	3b	Important changes to methods after trial commencement (such as eligibility criteria), with reasons	Page 5
Participants	4a	Eligibility criteria for participants	Page 5, Figure 1
	4b	Settings and locations where the data were collected	Page 4,5
Interventions	5	The interventions for each group with sufficient details to allow replication, including how and when they were actually administered	Page 4,5,6; Suppl. file 2
Outcomes	6a	Completely defined pre-specified primary and secondary outcome measures, including how and when they were assessed	Page 6-10
	6b	Any changes to trial outcomes after the trial commenced, with reasons	none
Sample size	7a	How sample size was determined	Page 10
	7b	When applicable, explanation of any interim analyses and stopping guidelines	none
Randomisation:			
Sequence generation	8a	Method used to generate the random allocation sequence	Page 2,4
	8b	Type of randomisation; details of any restriction (such as blocking and block size)	Page 2,4
Allocation concealment mechanism	9	Mechanism used to implement the random allocation sequence (such as sequentially numbered containers), describing any steps taken to conceal the sequence until interventions were assigned	Page 4
Implementation	10	Who generated the random allocation sequence, who enrolled participants, and who assigned participants to interventions	Page 4

Blinding	11a	If done, who was blinded after assignment to interventions (for example, participants, care providers, those assessing outcomes) and how	Page 2,3,4,5
	11b	If relevant, description of the similarity of interventions	Page 5,6
Statistical methods	12a	Statistical methods used to compare groups for primary and secondary outcomes	Page 10; Suppl file 1
	12b	Methods for additional analyses, such as subgroup analyses and adjusted analyses	Page 10,12, Suppl file 1
**Results**			
Participant flow (a diagram is strongly recommended)	13a	For each group, the numbers of participants who were randomly assigned, received intended treatment, and were analysed for the primary outcome	Figure 1; Table 3,4,5; Page 2, 11
	13b	For each group, losses and exclusions after randomisation, together with reasons	Figure 1 ; Table 3; Page 11,17
Recruitment	14a	Dates defining the periods of recruitment and follow-up	Page 4,5,7
	14b	Why the trial ended or was stopped	Page 7
Baseline data	15	A table showing baseline demographic and clinical characteristics for each group	Tables 3 and 4
Numbers analysed	16	For each group, number of participants (denominator) included in each analysis and whether the analysis was by original assigned groups	Table 4,5,6 ; Suppl file 1 ; Figure 1 ; Page 11-16
Outcomes and estimation	17a	For each primary and secondary outcome, results for each group, and the estimated effect size and its precision (such as 95% confidence interval)	Table 5, 6; Suppl file 1; Page 12-16
	17b	For binary outcomes, presentation of both absolute and relative effect sizes is recommended	Table 5, 6; Suppl file 1; Page 12-16
Ancillary analyses	18	Results of any other analyses performed, including subgroup analyses and adjusted analyses, distinguishing pre-specified from exploratory	Suppl file 1
Harms	19	All important harms or unintended effects in each group (for specific guidance see CONSORT for harms)	Table 5
**Discussion**			
Limitations	20	Trial limitations, addressing sources of potential bias, imprecision, and, if relevant, multiplicity of analyses	Page 10, 16-

Krones T, *et al. BMJ Support Palliat Care* 2022; 12:e411–e423. doi: 10.1136/bmjspcare-2017-001489

			19
Generalisability	21	Generalisability (external validity, applicability) of the trial findings	Page 16-19
Interpretation	22	Interpretation consistent with results, balancing benefits and harms, and considering other relevant evidence	Page 16-19
**Other information**			
Registration	23	Registration number and name of trial registry	Page 4
Protocol	24	Where the full trial protocol can be accessed, if available	Page 4
Funding	25	Sources of funding and other support (such as supply of drugs), role of funders	Page 4

*We strongly recommend reading this statement in conjunction with the CONSORT 2010 Explanation and Elaboration for important clarifications on all the items. If relevant, we also recommend reading CONSORT extensions for cluster randomised trials, non-inferiority and equivalence trials, non-pharmacological treatments, herbal interventions, and pragmatic trials. Additional extensions are forthcoming: for those and for up to date references relevant to this checklist, see www.consort-statement.org.

Additional features for pragmatic trials (Zwarenstein et al. Improving the reporting of pragmatic trials: an extention of the CONSORT statement. BMJ 2006; 337)

Addition to 2 Background
Describe the health or health service problem that the intervention is intended to address and other interventions that may commonly be aimed at this problem
Page 3-4

Addition to 3 Participants
Eligibility criteria should be explicitly framed to show the degree to which they include typical participants and/or, where applicable, typical providers (eg, nurses), institutions (eg, hospitals), communities (or localities eg, towns) and settings of care (eg, different healthcare financing systems)
Page 5-6, Table 3

Addition to 4 Interventions
Describe extra resources added to (or resources removed from) usual settings in order to implement intervention. Indicate if efforts were made to standardise the intervention or if the intervention and its delivery were allowed to vary between participants, practitioners, or study sites
Describe the comparator in similar detail to the intervention
Page 4,7

Addition to 6 Chosen Outcomes
Explain why the chosen outcomes and, when relevant, the length of follow-up are considered important to those who will use the results of the trial
Page 7-10

Krones T, *et al. BMJ Support Palliat Care* 2022; 12:e411–e423. doi: 10.1136/bmjspcare-2017-001489

Addition to 7 Sample Size
If calculated using the smallest difference considered important by the target decision maker audience (the minimally important difference) then report where this difference was obtained
n.a.

Addition to 11 Blinding
If blinding was not done, or was not possible, explain why
Page 4-5 why blinding was possible but not regarding full observer blinding, discussion 18-19

Addition to 13 Participant flow
The number of participants or units approached to take part in the trial, the number which were eligible, and reasons for non-participation should be reported
Table 3, Table 4, figure 1

Addition to 21 Generalisability
Describe key aspects of the setting which determined the trial results. Discuss possible differences in other settings where clinical traditions, health service organisation, staffing, or resources may vary from those of the trial
Page 16-20
.

Krones T, *et al. BMJ Support Palliat Care* 2022; 12:e411–e423. doi: 10.1136/bmjspcare-2017-001489
